# Methylation-regulated tumor suppressor gene *PDE7B* promotes HCC invasion and metastasis through the *PI3K/AKT* signaling pathway

**DOI:** 10.1186/s12885-024-12364-w

**Published:** 2024-05-22

**Authors:** Yuanxiao Du, Yuqiu Xu, Xuefeng Guo, Chao Tan, Xiaonian Zhu, Guoyu Liu, Xiao Lyu, Chunhua Bei

**Affiliations:** 1 Department of Epidemiology and Health Statistics, School of Public Health, Guilin Medical University, Huan Cheng North 2nd Road 109, Guilin, Guangxi 541004 China; 2 Guangxi Key Laboratory of Environmental Exposomics and Entire Lifecycle Heath, Guangxi Health Commission Key Laboratory of Entire Lifecycle Health and Care, School of Public Health, Guilin Medical University, Guilin, China

**Keywords:** *PDE7B*, Hepatocellular carcinoma, DNA methylation, *PI3K/AKT*

## Abstract

**Background:**

Hepatocellular carcinoma (HCC) has a high mortality rate, and the mechanisms underlying tumor development and progression remain unclear. However, inactivated tumor suppressor genes might play key roles. DNA methylation is a critical regulatory mechanism for inactivating tumor suppressor genes in HCC. Therefore, this study investigated methylation-related tumor suppressors in HCC to identify potential biomarkers and therapeutic targets.

**Methods:**

We assessed genome-wide DNA methylation in HCC using whole genome bisulfite sequencing (WGBS) and RNA sequencing, respectively, and identified the differential expression of methylation-related genes, and finally screened phosphodiesterase 7B (*PDE7B*) for the study. The correlation between *PDE7B* expression and clinical features was then assessed. We then analyzed the changes of *PDE7B* expression in HCC cells before and after DNA methyltransferase inhibitor treatment by MassArray nucleic acid mass spectrometry. Furthermore, HCC cell lines overexpressing *PDE7B* were constructed to investigate its effect on HCC cell function. Finally, GO and KEGG were applied for the enrichment analysis of *PDE7B*-related pathways, and their effects on the expression of pathway proteins and EMT-related factors in HCC cells were preliminarily explored.

**Results:**

HCC exhibited a genome-wide hypomethylation pattern. We screened 713 hypomethylated and 362 hypermethylated mCG regions in HCC and adjacent normal tissues. GO analysis showed that the main molecular functions of hypermethylation and hypomethylation were “DNA-binding transcriptional activator activity” and “structural component of ribosomes”, respectively, whereas KEGG analysis showed that they were enriched in “bile secretion” and “Ras-associated protein-1 (Rap1) signaling pathway”, respectively. *PDE7B* expression was significantly down-regulated in HCC tissues, and this low expression was negatively correlated with recurrence and prognosis of HCC. In addition, DNA methylation regulates *PDE7B* expression in HCC. On the contrary, overexpression of *PDE7B* inhibited tumor proliferation and metastasis in vitro. In addition, *PDE7B*-related genes were mainly enriched in the *PI3K/ATK* signaling pathway, and *PDE7B* overexpression inhibited the progression of *PI3K/ATK* signaling pathway-related proteins and EMT.

**Conclusion:**

*PDE7B* expression in HCC may be regulated by promoter methylation. *PDE7B* can regulate the EMT process in HCC cells through the *PI3K/AKT* pathway, which in turn affects HCC metastasis and invasion.

**Supplementary Information:**

The online version contains supplementary material available at 10.1186/s12885-024-12364-w.

## Introduction

Primary liver cancer, one of the most prevalent malignancies in the digestive system. Among them, hepatocellular carcinoma (HCC) accounts for 75–85%, and the mortality rate ranks second among malignant tumors. Approximately 422,100 people die of liver cancer each year [1], creating severe economic and social burdens. The early detection of HCC and comprehensive treatment, primarily surgical resection, can improve treatment outcomes. However, the five-year survival rate of patients with advanced HCC and metastasis is less than 10% [2]. For patients with HCC, recurrence and metastasis are the main causes of death and the most difficult aspects to treat, partially because the exact mechanisms underlying HCC metastasis have not yet been fully elucidated.

Epigenetics refers to reversible and heritable changes in gene function that occur without DNA sequence alterations. Epigenetic regulation is a critical role in disease onset and progression, and restoring the epigenetic genome can prevent disease progression [3]. DNA methylation, an important epigenetic regulatory pathway, is catalyzed by DNA methyltransferases. The active methyl group is transferred from S-adenosylmethionine to the 5-carbon position of cytosine to form 5-methylcytosine, changing the DNA conformation, stability, and protein interactions, thereby regulating gene expression [4]. Abnormally high methylation of CpG islands in the promotor regions of tumor suppressor genes is an important gene inactivation mechanism in cancer cells [5–7]. For instance, demethylation therapy, which targets the hypermethylation of tumor suppressor genes, prolongs the overall survival of patients with advanced tumors [8]. Currently, 5-aza-CdR is the most widely used methylation inhibitor, which has good prospects for clinical application since it causes demethylation of most highly-methylated tumor-suppressor genes, inhibiting tumor progression in vitro and in vivo [9]. In recent decades, molecular targeted therapies have become the focus of cancer treatment, and targeted therapy remains the primary treatment for patients with advanced HCC. Liu et al. Ras-associated nuclear proteins are in a state of high expression and promoter hypomethylation in HCC and portend a poor survival prognosis [10]. Additionally, Chalasani et al. found that the combination of DNA methylation markers (*HOXA1*, *EMX1*, *TSPYL5* and* B3GALT6*) and protein markers (AFP and AFP-L3) could improve the accuracy of detecting early HCC [11]. Therefore, exploring the regulatory mechanisms of DNA methylation in HCC metastasis could result in new molecular targets for targeted therapy, improving the treatment’s clinical efficacy and survival prognosis of patients with HCC.

Phosphodiesterase 7B (*PDE7B*) is a member of the phosphodiesterase (PDE) family that hydrolyzes intracellular cyclic adenosine monophosphate (cAMP) and cyclic guanosine monophosphate (cGMP). *PDE7B* participates in various physiological processes by hydrolyzing cAMP and cGMP, such as cell proliferation, differentiation, inflammation, and metabolic functions [12]. The *PDE7* family consists of two subtypes, *PDE7A* and *PDE7B*, which have high affinity and specificity for cAMP [13]. *PDE7B*, located on chromosome 6q23-24, has a protein kinase A phosphorylation site [14] and regulates the cAMP signal transduction pathway by reducing intracellular cAMP concentrations through hydrolysis. Earlier studies have found that *PDE7B* acts in cells to reduce intracellular cAMP concentration through hydrolysis, as shown by Brooks et al [15]. Additionally, Zhang et al. found that *PDE7B* plays an oncogenic role in regulating cell growth and tumor development by knocking down *PDE7B* and regulating the cellular cAMP concentration in triple-negative breast cancer cells [16]. However, more recent studies have shown the opposite, indicating that *PDE7B* is downregulated in renal tumors and has a tumor-suppressive role [17, 18]. Specifically, Sun et al. reported that *PDE7B* was downregulated in renal clear cell carcinoma, and knocking down *PDE7B* boosted the growth, metastasis and infiltrative capacities of renal clear cell carcinoma cells [17]. Additionally, Liu et al. reported lower *PDE7B* expression in osteosarcoma tissues than in normal tissues and that *PDE7B* overexpression inhibited the proliferation, migration, and invasion abilities of osteosarcoma cells [18]. Therefore, the function of *PDE7B* may differ among tumor tissues and cells. However, how *PDE7B* affects the development of HCC and its mechanism of action has not been investigated.

In addition, invasion and metastasis are the main features of malignant progression of tumors, and EMT is one of its early markers and an important direction in the current research of malignant progression of tumors. EMT is a process in which the epithelial cells lose their cellular polarity and adhesion and acquire a mesenchymal cell phenotype such as a higher ability to migrate and invade, resistance to apoptosis, and the ability to degrade extracellular matrix [19, 20]. Cells undergoing the EMT process also undergo changes at the molecular level, which are mainly characterized by the downregulation of the expression of epithelial markers (*E-cadherin*, *β-catenin*, *ZO-1*, etc.) and the upregulation of mesenchymal markers (*N-cadherin*, *Vimentin*, etc.) [21]. Furthermore, zinc finger proteins such as the transcription factor *Snail* can promote the EMT process by inhibiting the transcription of epithelial markers such as *E-cadherin* and upregulating the expression of mesenchymal cell markers such as *Vimentin*. And a large number of studies have shown that the EMT process in hepatocellular carcinoma is associated with the invasive metastatic ability of hepatocellular carcinoma cells [22, 23]. Therefore, the potential mechanism of action of *PDE7B* on HCC invasive migration can be determined by observing its effect on the expression of relevant proteins during EMT.

Currently, only a few studies have reported on the expression and role of *PDE7B* in HCC. Therefore, this study used whole-genome methylation sequencing and transcriptome sequencing in HCC and adjacent normal tissues to investigate methylation-related tumor suppressors in HCC and identify potential biomarkers and therapeutic targets.

## Materials and methods

### Patients and tissue samples

Paired HCC and adjacent non-tumor tissues were collected for immunohistochemistry test from the Affiliated Hospital of Guilin Medical University. A total of 84 paired tissue samples were placed in liquid nitrogen for long-term storage shortly after surgery, from HCC patients who received no any chemotherapy or radiation therapy other than surgery. This find out about was once performed beneath knowledgeable consent from all sufferers and the endorsement of the Ethics Committee of the First Affiliated Hospital of Guilin Medical University (Guangxi, China), and it used to be carried out following the Helsinki Declaration.

Clinical information for these three patients was acquired from the hospital’s electronic medical record system. A pathological diagnosis of HCC was made in all patients, and all tested positive for hepatitis B virus (HBV). The mean age of these patients was 54.67 years. The clinical stage was I or II, and the case grading was around medium (Supplementary Table 1).

### Genomic DNA extraction and WGBS library construction

Genomic DNA (gDNA) retrieved from samples of three sets of paired HCC tissues and normal tissues was used to prepare the library. For WGBS library construction, it is a combination of heavy sulfite treatment and high-throughput sequencing, using heavy sulfite treatment of sample DNA to convert all unmethylated cytosine (C) to uracil (U) on the genome, and unmethylated cytosine (C) to thymine (T) on the genome after PCR amplification, and then combined with high-throughput sequencing technology to map single-base resolution whole genomic DNA methylation profiles at single-base resolution.

### WGBS data processing

Data screening is the elimination of polluted, sequenced junction and low-quality base over-represented reads out of the original data. Any reads that meet the following conditions are considered low quality reads: (1) more than 10% unknown bases and (2) more than 10% bases with quality below 20. Next the clean data were plotted onto the standard reference genome by BSMAP and the plotting rate and bisulfite transition rate were measured for an individual sample.

### Methylation level and differentialy methylated region (DMR) detection

Methylation level was determined by dividing the number of reads covering each mC by the total reads covering that cytosine [24], which was also equal the mC/C ratio at each reference cytosine [25]. The formula is showed as following figure:

$$R{m_{averag{e_{_{}}}}} = \frac{{N{m_{all}}}}{{N{m_{all}} + Nn{m_{all}}}}*100\%$$

Nm represents the reads numberof mC, whileNnm represents the reads number of nonmethylationreads.

The criterion established by DMR was a more than 2-fold difference in methylation levels of at least 5 CpG loci between the two groups with *P* < 0.05. When a 2-fold difference in methylation levels of gene regions between the starting region of one segment of DMR to the end point of another segment of DMR was also encountered with a P value < 0.05, then a continuous segment of DMR was combined.

### RNA sequencing

A certain amount of genomic DNA is taken for fragmentation. The DNA fragments are size-selected. The reaction system is configured, and the reaction program is set up to repair the end of DNA and add A base to the 3 ‘end. The adaptors ligation reaction system is configured, and the reaction program is set up to connect the adaptors with the DNA fragments. The reaction system is configured, and the reaction program is set up for Bisulfite treatment and purification. The PCR reaction system and program are configured and set up to amplify the product. The corresponding library quality control protocol will be selected depending upon product requirements.The reaction system and program for circularization are subsequently configured and set up. Circular DNA molecules are circularly amplified into DNA nanoballs (DNBs), which are then sequenced using nanochip technology and combinatorial probe synthesis.

### Identification of differentially expressed methylated genes

DMR data were downloaded from BGI online (https://biosys.bgi.com/). Then, the annotation information was sorted, and the duplicates were removed, resulting in two gene sets with hypermethylated and hypomethylated modifications. The definition of differentially expressed genes (DEGs) was determined by the criterion of *P* < 0.05. The criterion for downregulated genes was a log2FC value of ≤–1, while the criterion for upregulated genes was a log2FC value of ≥ 1. The crossover genes between the downregulated and hypermethylated genes were considered hypomethylated, low-expression genes. In contrast, crossover genes between the upregulated and hypermethylated genes were considered hypermethylated, highly expressed genes. Venn and volcano diagrams were plotted using the ggplot2 package on the Venn diagram website (https://bioinformatics.psb.ugent.be/webtools/Venn/) and in R (R Core Team, Vienna, Austria), respectively.

### Cell lines

Human hepatocytes (L02) had been bought from the Cell Library (Chinese Academy of Sciences, Shanghai, China), while hepatic tumor cell lineages (SMMC-7721, HCC-97 H, Huh-7, SK-Hep-1) had been bought from ATCC (Manassas, VA, USA). Throughout the experiments, all cells were cultured in an incubator (37 °C, 5% CO_2_).

### Immunohistochemical staining

Briefly, tissue sections were deparaffinized, rehydrated, and subjected to antigen retrieval by boiling in citrate buffer (pH = 6, ZSGB-Bio, Beijing, China). The sections were then incubated overnight with anti-protein X antibody (Proteintech, Wuhan, China) at 4 °C. Following that, the sections were washed 3 times with PBS (ZSGB-Bio, Beijing, China) and incubated with secondary antibody (ZSGB-Bio, Beijing, China) for 1 h at ambient temperature. After three washes with PBS (ZSGB-Bio, Beijing, China), the sections were incubated with streptavidin-biotin-peroxidase complex (ZSGB-Bio, Beijing, China) for 1 h. Finally, the sections were visualized with domain antibodies and counterstained with hematoxylin (sigma inc., USA). Negative controls were prepared by omitting the primary antibody. Immunohistochemical results were scored by two independent pathologists using a semi-quantitative system based on staining intensity and percentage of positively stained cells.

### Quantitative reverse-transcription polymerase chain reaction (qRT-PCR)

Total RNA of the cell lines was extracted using the precipitation approach and the concentration was determined using NanoDrop2000 (ThermoFisher Scientific). Then reverse RNA to cDNA by using reverse transcription reagent (Takara, code RR047A), accompanied via way of the addition of TB Green Mixture (Takara, code RR820A) in accordance to the directions to begin the PCR reaction. *GAPDH* (BGI, China) was set as an internal reference control. The primers utilized in the work are listed below:

*PDE7B* Forward primer: 5’-TGGGAGATATACGACTAAGGGGT-3’;

*PDE7B* Reverse primer: 5’-CGGAAGTCAATGAATGGGTAGG-3’;

*GAPDH* Forward primer: 5’-ACAACTTTGGTATCGTGGAAGG-3’;

*GAPDH* Reverse primer: 5’-GCCATCACGCCACAGTTTC-3’.

### CCK8 assay

To notice cell proliferation activity, cells were inoculated in 96-well plates at 5 × 10^3^ cells/well, with 5 replicate wells for every crew of cells. After 12 h, 24 h, 48 h and 72 h of cultivation, the cells were handled with 10ul CCK8 (MedChemExpress, USA) in 96-well plates and cultivated in an incubator for 2 h, followed by measurement of OD at 450 nm.

### Formation of cell colony

Cells were inoculated in six-well plates at 600 cells/well in 3 replicate wells for each cell group and incubated for 2 weeks. After washing the cells 3 times with PBS, fixed with 4% paraformaldehyde (Solarbio Technology Co., Ltd., Beijing, China) for 30 min and dyed with crystal violet (Solarbio Science & Technology Co., Ltd., Beijing, China) for 30 min. Following ultra-pure water washing, the cell colonies were pictured, tallied and analyzed.

### Cell scratch detection

Cells could be scratched 6 ∼ 8 h after transfection. That is, the cells were scratched vertically along a straightedge with a 10 µL white tip on a monolayer of adherent cells, washed 2 ∼ 3 times with PBS and incubated by adding medium (Gibco, Thermo Fisher Scientific, Virginia, USA) containing no serum. The migration of cells at the identical scratch location at 0 h and 24 h was then recorded under the microscope.

### Migration and invasion assays

Transwell assays are used to detect cell migration and invasion ability. All 8 μm pore size chambers need to be activated by adding serum-free medium (Gibco, Thermo Fisher Scientific, Virginia, USA) in an incubator for 30 min. For the invasion assay, 100ul of matrixgel (Corning, USA) was added to the chambers for 2 h before inoculation and excess matrix gel was removed. Then 200 ul of serum-free medium cell suspension at a density of 2 × 10^4^ and 600 ul of medium containing 10% serum (Tianhang Biotechnology Co., Zhejiang, China) were added to the upper and lower chambers, respectively. After 30 h of incubation, the cells were fixed and stained using 4% paraformaldehyde (Solarbio Science & Technology Co., Ltd., Beijing, China) and crystalline violet stain (Solarbio Science & Technology Co., Ltd., Beijing, China), respectively. Excess stain was washed off with PBS, and cells in the upper chamber were wiped off and pictured and tallied below microscope.

### In vitro cell line demethylation assay

Cells in logarithmic growth phase and with good growth status were prepared into a cell suspension and diluted to a concentration of 1.5 × 10^5^ cells/ml. Then, 1mL of diluted cell suspension was added to each well of a six-well plate, along with an additional 1mL of complete culture medium. Control and treatment groups were labeled and placed in a cell incubator for cultivation. After 24 h, 2mL of 0.1% dimethyl sulfoxide solution (Sigma inc., USA) was added to the control group, and 10µM working concentration of 5-azacytidine (Sigma inc., USA) was added to the treatment group. During the culturing process, cell morphology and growth rate were observed, and fresh drugs were replaced every 24 h. Control and treatment group cells were harvested for DNA and RNA extraction after treatment for 72 h.

### DNA methylation sequencing

Massarray DNA methylation quantitative detection employs matrix-assisted laser desorption/ionization time-of-flight mass spectrometry (MALDI-TOF MS) to detect and quantify methylated DNA sequences. The process involves the use of bisulfite treatment to convert unmethylated cytosine residues to uracil, leaving methylated cytosine residues intact. The converted DNA is then amplified using PCR and analyzed by mass spectrometry. The mass signal obtained is proportional to the number of copies of each DNA sequence, thus allowing quantification of the degree of methylation at individual CpG sites.

### Bioinformatics prediction and database analysis

RNA sequencing data of 33 cancers was downloaded from the Cancer Genome Atlas Program (TCGA). Perl (version 5.32.1, https://www.perl.org) scripts were used to extract gene expression matrices from the dataset, and R (version 4.1.0, https://mirrors.tuna.tsinghua.edu.cn/CRAN/) was used to analyze the differential expression of *PDE7B* in the 33 cancers. Differential analysis results were then visualized using the “ggplot2” package. Additional online datasets including ICGC-LIRI-JP, CNHPP, GSE14520, GSE102079, GSE107170 were also analyzed.

Methylation sequencing data of TCGA-LIHC cohort was downloaded from TCGA database. Perl scripts were used to extract gene methylation matrices from the dataset, and the differential methylation of *PDE7B* in liver cancer and the methylation differences at *PDE7B* methylation sites in liver cancer were analyzed with the R software. The genes related to *PDE7B* (Spearman correlation ≥ 0.3) were analyzed through the cBioPortal database. Then, the “clusterProfiler” R package was used to obtain Gene Ontology (GO) and Kyoto Encyclopedia of Genes and Genomes (KEGG) pathway information. And the results were visualized using the “ggplot2” package.

### Statistical analyses

The prognostic value was delineated with the aid of Kaplan-Meier survival curve which was checked for statistical significance based on log-rank test. Student’s t-test, one-way ANOVA, or Pearson chi-square test were utilized in this study to determine differences in all intergroup variables. The criterion for meaningful difference for all analyses was *P* < 0.05.

## Results

### DNA methylation characteristics and differentially methylated regions in HCC and adjacent normal tissue

WGBS analysis of three pairs of HCC and adjacent normal tissues yielded an average of 145.823 G of original data per sample. With reference to the human genome [19], clean reads and mapping rates were screened for each sample original data as illustrated in Table 1. Although the whole-gene hypomethylation status was present in both HCC tissues and paracancerous tissues (Supplementary material 1, Fig. 1), the proportion of genomic CpG site methylation in HCC tissues was 4.246%, which was slightly higher than that in paracancerous tissues (Table 1). In addition, an average of about 46,945,755 and 48135939.67 methylated cytosines (mC) were measured in each sample in both groups (Table 2). The human methylation profile includes three variable sequence states: CG, CHG, and CHH. Sequencing results showed that mCG accounted for the largest proportion of methylation types in both groups (Supplementary material 1, Fig. 2A, B). Furthermore the methylation levels varied in different regions, and it was observed that CG methylation levels were significantly higher in HCC than in paracancerous tissues in the gene regions (Supplementary material 1, Fig. 2C, D).

To reveal the potential functions of DMGs in HCC. 713 hypomethylated and 362 hypermethylated genes under mCG type in HCC tissues and adjacent normal tissues were screened from differentially methylated regions (DMRs). GO analysis revealed that we found that the major molecular function terms of hyper-DMGs and hypo-DMGs were “DNA-binding transcriptional activator activity” and “structural components of ribosomes”, respectively. The major cellular component terms for hyper-DMGs were “synaptic membrane composition” and “actin cytoskeleton,” whereas the major cellular component term for hypo-DMGs was “ribosome composition.” The main biological process terms for high and low DMGs were “embryonic organ development” and “cytoplasmic translation”, respectively (Supplementary material 1, Fig. 3A, B). Whereas KEGG analysis showed that high DMGs were mainly enriched in “bile secretion” and various other metabolic pathways, low DMGs were mainly enriched in “ribosomes,” “adherens junctions,” and “Ras-related protein-1 (Rap1) signaling pathway” (Supplementary material 1, Fig. 4A, B).

**Table 1:** Genomic methylation profile

Sample ID	mC (%)	mCG (%)	mCHG (%)	mCHH (%)
C1^a^	3.705	54.088	0.860	0.916
C2^a^	4.457	64.147	0.926	0.992
C3^a^	4.429	72.570	0.836	0.819
P1^b^	4.748	73.928	0.877	0.932
P2^b^	3.137	42.606	0.825	0.904
P3^b^	4.853	75.389	0.919	0.908
C^ac^	4.197 ± 0.666	63.602 ± 9.253	0.874 ± 0.047	0.909 ± 0.087
P^bc^	4.246 ± 0.962	63.974 ± 18.520	0.874 ± 0.047	0.915 ± 0.015
ALL^c^	4.222 ± 0.666	63.788 ± 13.095	0.874 ± 0.042	0.912 ± 0.056

Notes: ^a^Paraneoplastic tissue, ^b^Hepatocellular carcinoma tissue; ^c^Data are mean ± standard deviation

**Table 2:** Proportion of CG, CHG and CHH in the distribution of methylated C bases

Sample ID	mCG		mCHG		mCHH	
	*N* ^c^	%	*N* ^c^	%	*N* ^c^	%
C1^a^	41,790,212	94.272	476,562	1.075	2,062,475	4.653
C2^a^	46,708,686	92.799	692,944	1.377	2,931,324	5.824
C3^a^	46,694,978	93.868	726,390	1.46	2,324,248	4.672
P1^b^	47,915,941	92.972	679,326	1.318	2,942,882	5.71
P2^b^	35,290,706	92.487	504,073	1.321	2,362,538	6.192
P3^b^	47,761,272	93.39	759,053	1.484	2,621,474	5.126

Notes: ^a^Paraneoplastic tissue, ^b^Hepatocellular carcinoma tissue, ^c^mC number

### *PDE7B* was identified as a potential methylation-related tumor suppressor of HCC after data integration

Volcano plots were used to depict DEGs screened from the transcriptome sequencing data (Supplementary material 1, Fig. 5A); there were 711 genes up-regulated, while 757 genes were down-regulated. Besides, 27 hypermethylated/low-expression tumor suppressor genes were identified through the cross-tabulation analysis of the DMRs and DEGs (Supplementary material 1, Fig. 5B). Then the transcriptomic and clinical data of patients in the TCGA database were analyzed, and six DNA hypermethylation-regulated genes (*PDE7B, RORA, OBSCN, APCDD1, SLC22A1*, and *PNPLA7)* with different degrees of underexpression were found to be significantly underexpressed in HCC. By Kaplan-Meier survival analysis, we found that they all had a significant impact on the prognosis of HCC (Supplementary material 1, Fig. 5C). By reviewing the published literature, we chose PDE7B for further study to determine its effect on HCC.

### Validation the decreased expression of *PDE7B* and associated with HCC

**Fig. 1:**
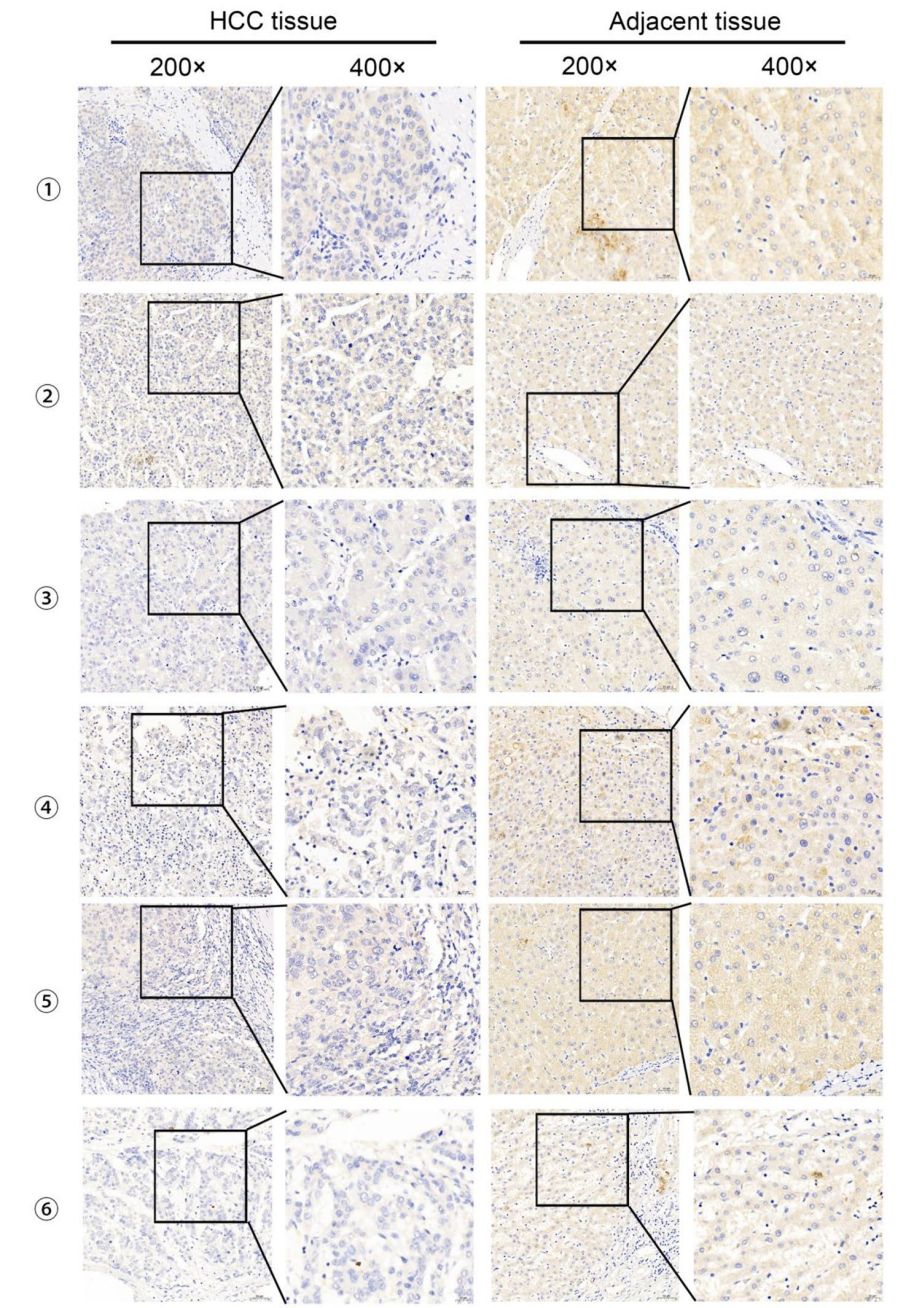
Immunohistochemical results of six cases of HCC tissues and their corresponding adjacent cancerous tissues

**Fig. 2:**
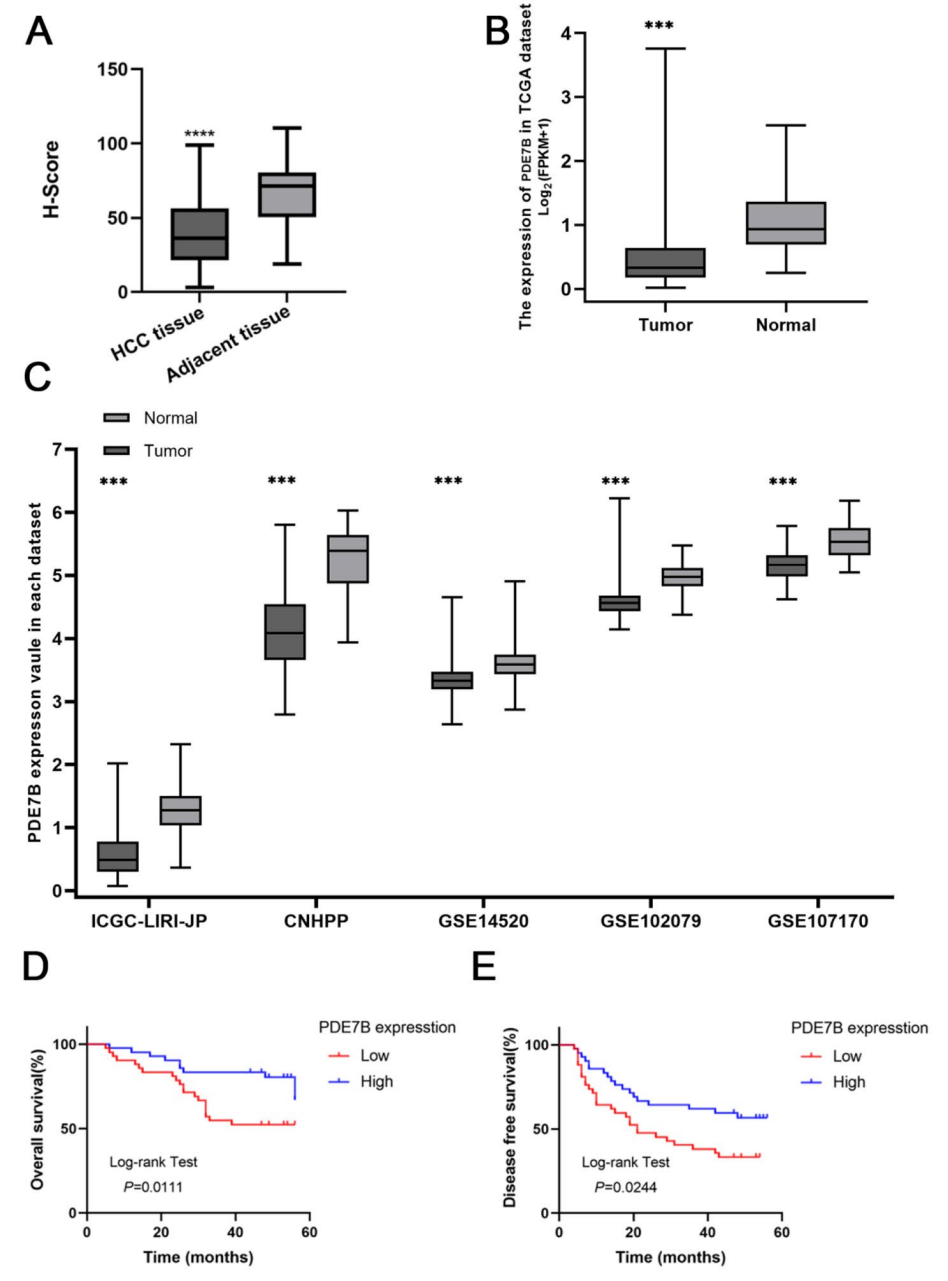
H-score for immunohistochemical detection of PDE7B expression results in HCC tissues and paracancerous tissues (**A**); Bioinformatics analysis of PDE7B expression in HCC and normal tissues in online datasets (TCGA, **B;** ICGC-LIRI-JP, CNHPP, GSE14520, GSE102079, GSE107170, **C**); analysis of overall survival (**D**) and Disease free survival (**E**) of HCC patients with high and low PDE7B expression. (***, *P* < 0.001)

The expression of *PDE7B* in tumor tissues (*n* = 84) and neighbouring normal tissues (*n* = 84) was detected using immunohistochemistry, and the tissues were obtained from the Affiliated Hospital of Guilin Medical College. It was found that decreased expression of *PDE7B* in HCC tissues compared to adjacent normal tissues (Figs. 1 and 2A). *PDE7B* was mainly expressed in the cytoplasm, which was generally consistent with the results of the The Human Protein Atlas database. The results of paired t-test also showed that *PDE7B* in HCC tissues had a significantly lower H-Score than that of adjacent normal tissues (Fig. 2A). Moreover, on the online dataset (TCGA-LIHC, ICGC-LIRI-JP, CNHPP, GSE14520, GSE102079 and GSE107170) for HCC analysis, the expression of PDE7B in tumors was also generally lower than that in normal tissues (Fig. 2B, C).

Based on the *PDE7B* immunohistochemistry results, we divided 84 HCC patients into *PDE7B* high expression and low expression groups. It was found that *PDE7B* expression was associated with clinical characteristics, including serum Alpha-fetoprotein (AFP) level and recurrence (Tables 3 and 4). Kaplan-Meier results showed that the expression of *PDE7B* affected the overall survival (OS) and Disease free survival (DFS) of HCC patients. Log-rank test revealed that the OS and DFS time of patients in the low *PDE7B* expression group were significantly lower than those in the high expression group (Fig. 2D, E).The prognostic value of *PDE7B* in HCC patients was further assessed by cox regression analysis. It was showed that low *PDE7B* expression was an independent risk factor of HCC(Tables 5 and 6).

**Table 3:** Association between the expression of PDE7B and clinicopathological features of HCC

Variable	*n*	PDE7B expression		χ^2^	*P*
		High(*n* = 42)	low(*n* = 42)		
Age at onset of diagnosis (years)					
< 60	64	31	33	0.263	0.608
≥ 60	20	11	9		
Gender					
Male	75	37	38	0.000	1.000
Female	9	5	4		
Tumor size(cm)					
< 5	53	29	24	1.278	0.258
≥ 5	31	13	18		
Number of tumors					
Single shot	74	36	38	0.114	0.736
Multi-incidence (≥ 2)	10	6	4		
Pathological grading					
I+II	62	32	30	0.246	0.620
III	22	10	12		
Clinical Staging					
I+II	83	42	41	--	1.000
III + IV	1	0	1		
T Staging					
T1 + T2	82	42	40	--	0.494
T3 + T4	2	0	2		
Is cirrhosis of the liver					
Yes	77	37	40	0.623	0.430
No	7	5	2		
Number of cirrhotic nodules					
Single shot	9	4	5	0.000	1.000
Multi-incidence (≥ 2)	75	38	37		
Whether relapse					
Yes	32	18	28	4.805	0.028
No	52	24	14		
Whether the tumor envelope is intact					
Yes	37	21	16	1.208	0.272
No	47	21	26		
HBsAg					
Positive	68	35	33	0.309	0.578
Negative	16	7	9		
HBcAb					
Positive	76	38	38	0.000	1.000
Negative	8	4	4		
AntiHCV					
Positive	1	1	0	--	1.000
Negative	83	41	42		
TB (µmol/L)					
< 17.1	62	29	33	0.985	0.321
≥ 17.1	22	13	9		
ALT (U/L)					
< 40	47	25	22	0.435	0.510
≥ 40	37	17	20		
ALB (g/dl)					
< 4.0	19	13	6	3.333	0.068
≥ 4.0	65	29	36		
AFP (ug/L)					
< 400	53	31	22	4.141	0.042
≥ 400	31	11	20		
GGT (U/L)					
< 50	42	24	18	1.714	0.190
≥ 50	42	18	24		

**Abbreviations**: HBsAg, hepatitis B surface antigen; HBcAb, hepatitis B core antibody; AntiHCV, hepatitis C virus antibody; TB, total bilirubin; ALT, alanine transaminase; ALB, albumin; AFP, alpha-fetoprotein; GGT, gamma-glutamyl transferase

**Table 4:** Multivariable logistic regression analysis of PDE7B expression in HCC

Variable	B	SE	Waldχ^2^	*P*	OR	95% CI for OR
Recurrence	1.057	0.477	4.901	0.027	2.877	1.129 ∼ 7.331
ALB (g/dl)	1.000	0.583	2.948	0.086	2.720	0.868 ∼ 8.522
AFP (ug/L)	0.988	0.495	3.987	0.046	2.687	1.018 ∼ 7.089

**Abbreviations**: ALB, albumin; AFP, alpha-fetoprotein

**Table 5:** Univariate and multifactor COX regression analysis of OS in HCC

Variable	Univariable analysis			multivariable analysis		
	HR	95% CI	*P*	HR	95% CI	*P*
PDE7B expression	2.662	1.207 ∼ 5.869	0.015	2.449	1.129 ∼ 5.529	0.024
Age at onset of diagnosis (years)	1.288	0.569 ∼ 2.914	0.543			
Gender	3.670	0.499 ∼ 26.993	0.202			
Tumor size(cm)	1.562	0.749 ∼ 3.261	0.235			
Number of tumors	1.560	0.593 ∼ 4.106	0.368			
Pathological grading	2.015	0.950 ∼ 4.275	0.068	1.760	0.810 ∼ 3.827	0.154
Clinical Staging	2.954	0.400 ∼ 21.845	0.289			
T Staging	2.960	0.699 ∼ 12.534	0.141			
Cirrhosis of the liver	3.074	0.418 ∼ 22.601	0.270			
Number of cirrhotic nodules	3.792	0.515 ∼ 27.946	0.191			
Whether the tumor envelope is intact	2.181	0.992 ∼ 4.799	0.053	1.746	0.769 ∼ 3.962	0.183
HBsAg	0.945	0.381 ∼ 2.343	0.903			
HBcAb	0.125	0.179 ∼ 1.232	0.125			
AntiHCV	0.670	0.00 ∼ 53483.37	0.670			
TB	1.297	0.591 ∼ 2.850	0.517			
ALT	1.237	0.597 ∼ 2.565	0.567			
ALB	1.507	0.429 ∼ 2.609	0.904			
AFP	1.102	0.520 ∼ 2.334	0.779			
GGT	1.559	0.744 ∼ 3.267	0.239			

**Abbreviations**: HBsAg, hepatitis B surface antigen; HBcAb, hepatitis B core antibody; AntiHCV, hepatitis C virus antibody; TB, total bilirubin; ALT, alanine transaminase; ALB, albumin; AFP, alpha-fetoprotein; GGT, gamma-glutamyl transferase

**Table 6:** Univariate and multifactorial COX regression analysis of DFS in HCC

Variable	Univariable analysis			multivariable analysis		
	HR	95% CI	*P*	HR	95% CI	*P*
PDE7B expression	1.939	1.071 ∼ 3.510	0.029	2.028	1.116 ∼ 3.685	0.020
Age at onset of diagnosis (years)	1.629	0.869 ∼ 3.054	0.128			
Gender	1.327	0.476 ∼ 3.703	0.588			
Tumor size(cm)	1.502	0.838 ∼ 2.692	0.172			
Number of tumors	1.776	0.826 ∼ 3.819	0.141			
Pathological grading	1.752	0.945 ∼ 3.251	0.075	1.868	1.003 ∼ 3.479	0.051
Clinical Staging	2.460	0.335 ∼ 18.089	0.377			
T Staging	2.001	0.482 ∼ 8.314	0.340			
Cirrhosis of the liver	2.307	0.559 ∼ 9.522	0.248			
Number of cirrhotic nodules	1.421	0.509 ∼ 3.965	0.502			
Whether the tumor envelope is intact	1.406	0.777 ∼ 2.543	0.260			
HBsAg	1.006	0.485 ∼ 2.086	0.987			
HBcAb	0.666	0.282 ∼ 1.571	0.353			
AntiHCV	0.048	0.000 ∼ 1055.202	0.552			
TB	0.922	0.477 ∼ 1.783	0.810			
ALT	1.118	0.627 ∼ 1.995	0.705			
ALB	1.041	0.517 ∼ 2.100	0.910			
AFP	1.010	0.555 ∼ 1.838	0.975			
GGT	1.516	0.845 ∼ 2.718	0.163			

**Abbreviations**: HBsAg, hepatitis B surface antigen; HBcAb, hepatitis B core antibody; AntiHCV, hepatitis C virus antibody; TB, total bilirubin; ALT, alanine transaminase; ALB, albumin; AFP, alpha-fetoprotein; GGT, gamma-glutamyl transferase

### *PDE7B* suppresses proliferation, migration, invasive ability of HCC cell

**Fig. 3:**
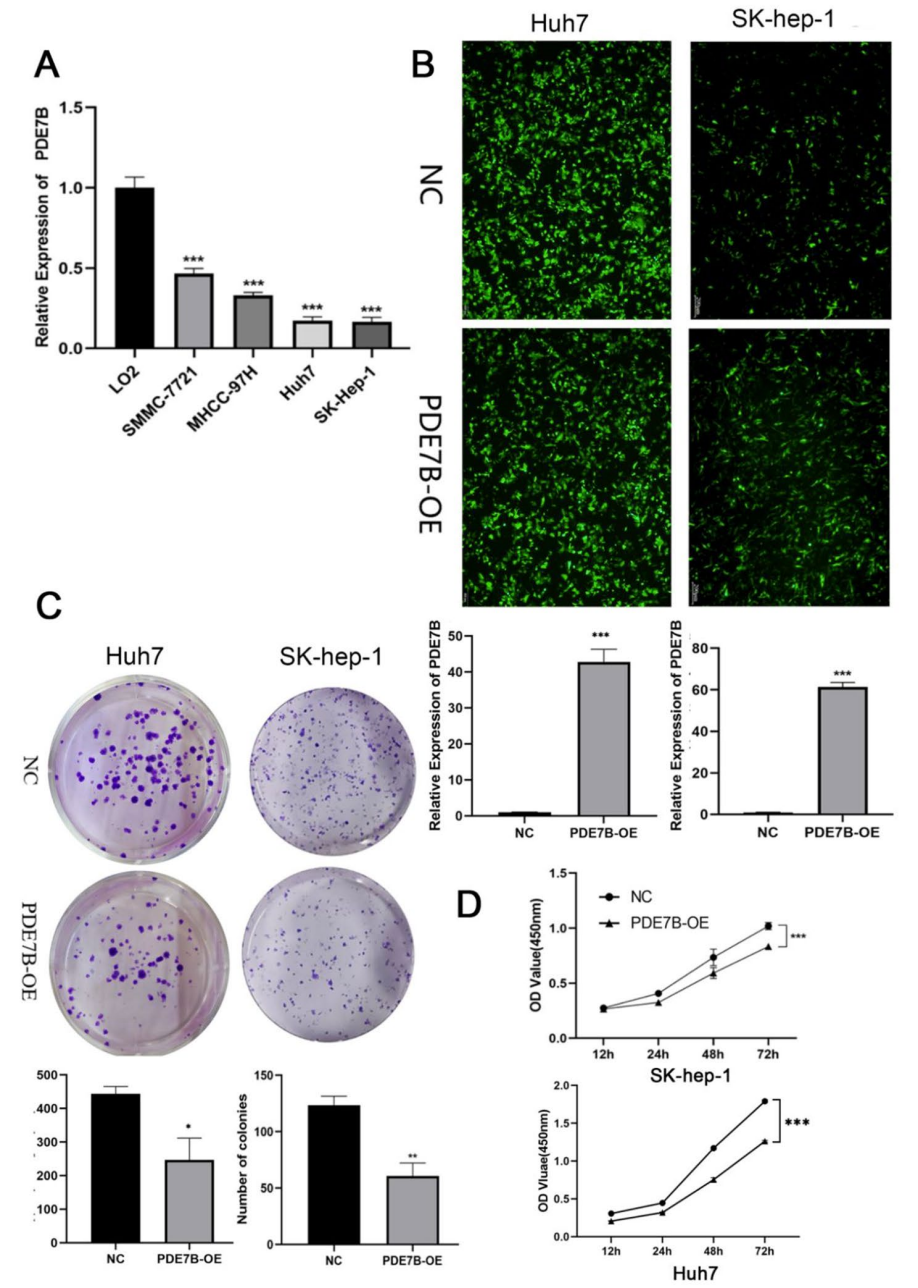
Expression levels of PDE7B in normal hepatocytes as well as hepatocarcinoma cell lines (**A**); observation of transfection efficiency of SK-hep-1 cells under fluorescence microscope and qRT-PCR to verify the transfection efficiency (**B**); plate clone-formation assay to detect the clonogenic ability of SK-hep-1 cells and Huh7 cells after PDE7B overexpression (**C**); CCK8 assay to detect the proliferation ability of SK-hep-1 cells and Huh7 cells after PDE7B overexpression (**D**). (*, *P* < 0.05;**, *P* < 0.01;***, *P* < 0.001)

The expression of *PDE7B* in SMMC-7721, MHCC-97 H, Huh7 and SK-hep-1 cells was detected by qRT-PCR. Compared with the expression of L02 in human HCC cell lines, the expression of *PDE7B* was reduced in SMMC-7721, MHCC-97 H, Huh7 and SK-hep-1. The two lowest PDE7B-expressing cell lines compared to L02 cellls were SK-hep-1 cellls and Huh7 cellls(Fig. 3A). Therefore, SK-hep-1 cells and Huh7 cells were screened to study the effect of PDE7B on the phenotype of HCC cells.

**Fig. 4:**
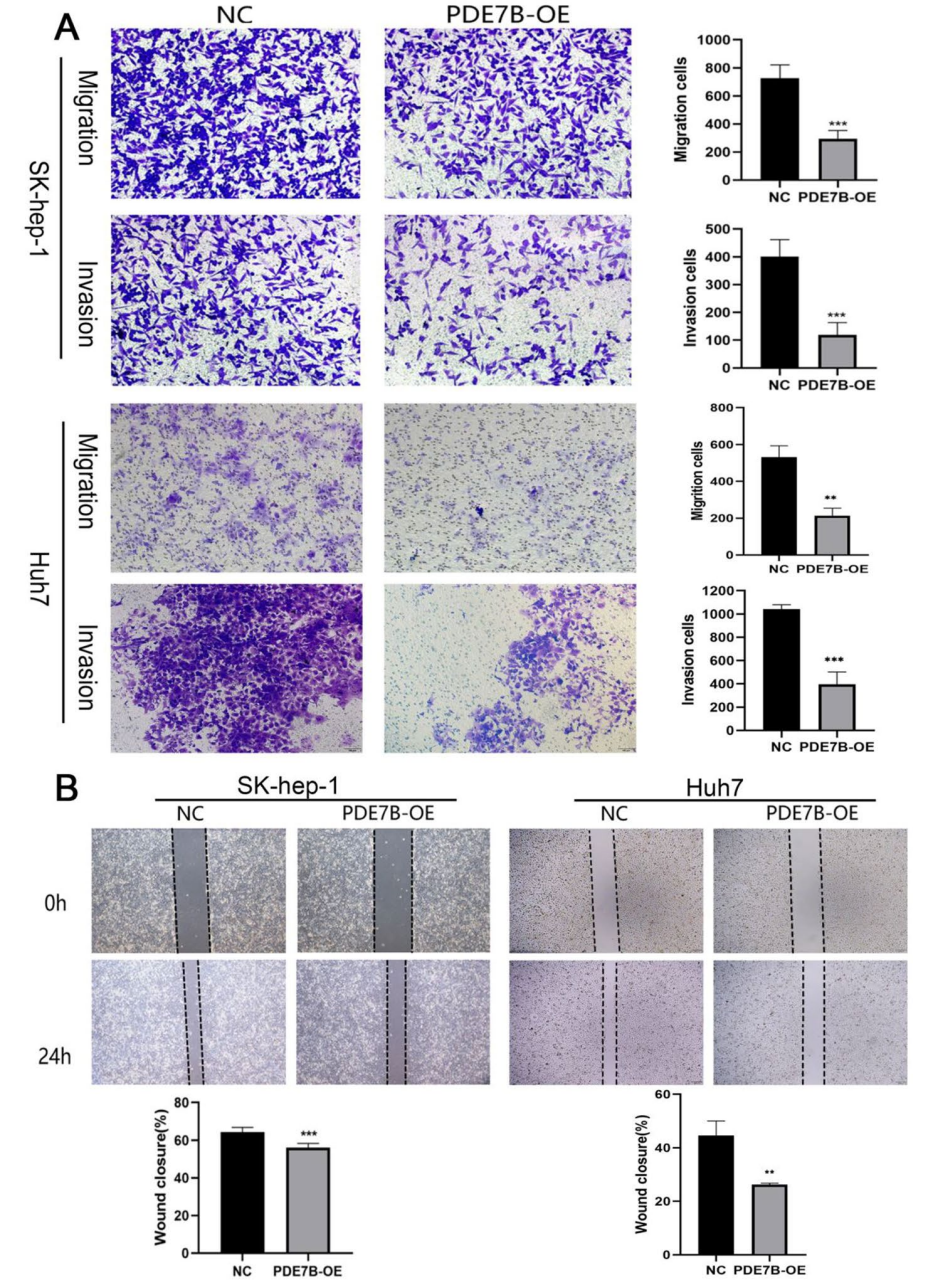
Transwell assay to detect the migration ability of SK-hep-1 cells and Huh7 cells after overexpression of PDE7B (**A**); scratch assay to detect the migration ability of SK-hep-1 cells and Huh7 cells after PDE7B overexpression (**B**). (*, *P* < 0.05;**, *P* < 0.01;***, *P* < 0.001)

To verify the effect of exogenous *PDE7B* overexpression (*PDE7B*-OE) on the biological function of HCC cell lines, SK-hep-1 cells and Huh7 cells were grouped and transfected with *PDE7B* gene overexpression plasmid and null plasmid, respectively (Fig. 3B). The expression of mRNA level of *PDE7B*-OE was significantly increased by qRT-PCR, and the mRNA expression levels were increased 60-fold in the SK-hep-1 cells and 42.7-fold in the Huh7 cells, respectively (Fig. 3B). The plate cloning assay showed that the number of cells formed by SK-hep-1 cell clones of *PDE7B*-OE was significantly reduced, and the same result was observed in Huh7 (Fig. 3C). CCK8 assay showed that the proliferative activity of SK-hep-1 and Huh7 of *PDE7B*-OE was significantly reduced (Fig. 3D). Transwell results showed that the number of cells penetrating into both matrix gel-free and matrix gel chambers in *PDE7B*-OE group was obviously weaker than the control (Fig. 4A). After scratch assay, the scratch healing rate of cells in *PDE7B*-OE group was found to be significantly lower than that in the control group (Fig. 4B). These results suggested that *PDE7B* inhibited the proliferation, migration and invasion of HCC cells.

### *PDE7B* expression was regulated by DNA methylation modifications in Human HCC Cell lines

**Fig. 5:**
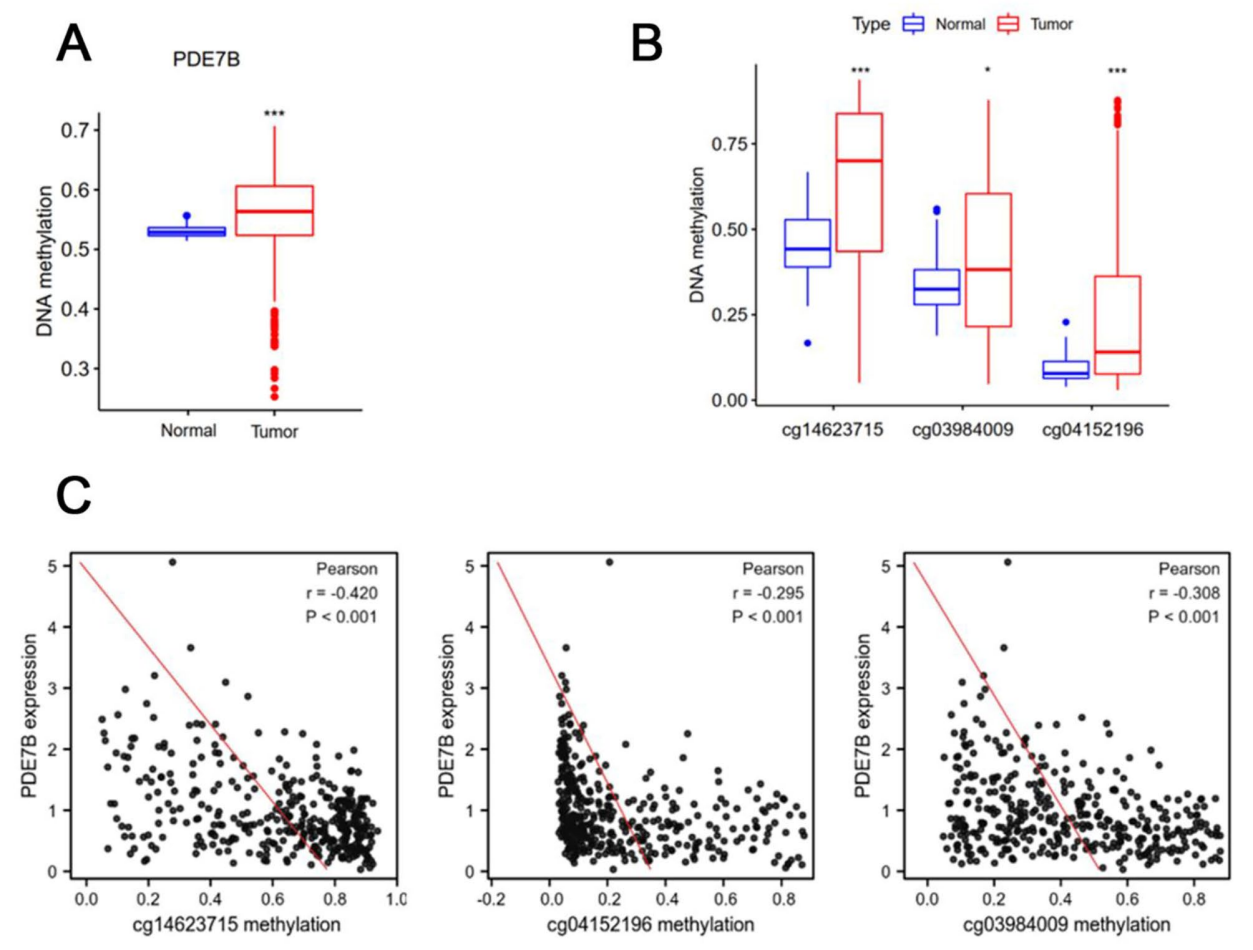
DNA methylation levels of PDE7B in HCC tissues from the TCGA database (**A**); significant differential DNA methylation sites for PDE7B in HCC tissues from the TCGA database (**B**); expression of PDE7B in the TCGA-LIHC cohort in relation to the methylation levels of cg sites (**C**). (*, *P* < 0.05;**, *P* < 0.01;***, *P* < 0.001;****, *P* < 0.0001)

**Fig. 6:**
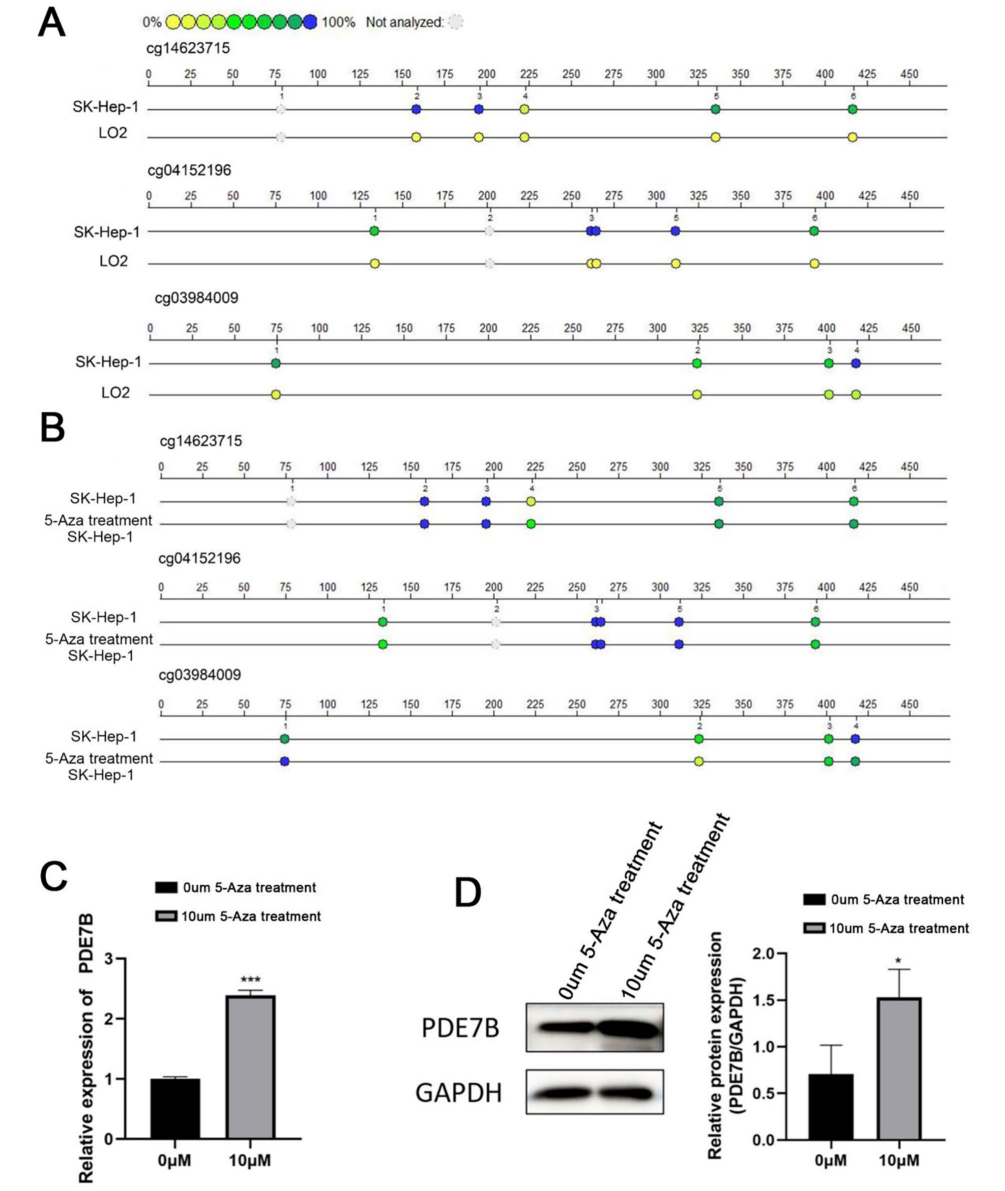
DNA methylation levels of PDE7B promoter CpG islands in LO2 and Sk-hep-1 cells detected by MassARRAY method (**A**). DNA methylation levels of *PDE7B* promoter CpG islands in sk-hep-1 cells before and after 5-Aza treatment by MassARRAY (**B**); mRNA and protein expression levels of *PDE7B* in hepatocarcinoma cell lines after 5-Aza treatment (**C, D**). (*, *P* < 0.05;**, *P* < 0.01;***, *P* < 0.001)

Methylome and transcriptome data of the LIHC cohort was downloaded from the TCGA database. The DNA methylation level of *PDE7B* in liver cancer was found to be significantly higher than that of controls (Fig. 5A), especially in cg14623715, cg3984009 and cg041521966 locus (Fig. 5B). Moreover, the degree of *PDE7B* expression was negatively linked to the three CpG loci mentioned above (Fig. 5C). In addition, it was showed that the above three locus methylation modifications were higher in SK-hep-1 than those in L02 cells (Fig. 6A). When SK-hep-1 was treated with 5-Aza (DNA methyltransferase inhibitor), it was found that the methylation level was reduced, with the highest decrease of 47.1% on the cg03984009 (Fig. 6B; Table 7), while mRNA and protein expression of *PDE7B* were significantly up-regulated (Fig. 6C, D).

**Table 7:** Methylation levels of HCC and normal hepatocytes before and after 5-Aza treatment

Gene name: PDE7B CpG	Sk-hep-1 DNA	Sk-hep-1 (5-Aza)	L02 DNA
PDE7B_CpG_1	0.81	1	0.12
PDE7B _CpG_2	0.51	0.27	0.22
PDE7B _CpG_3	0.61	0.61	0.37
PDE7B _CpG_4	0.96	0.89	0.35

### *PDE7B* inhibits EMT by regulating the *PI3K/AKT* signaling pathway

**Fig. 7:**
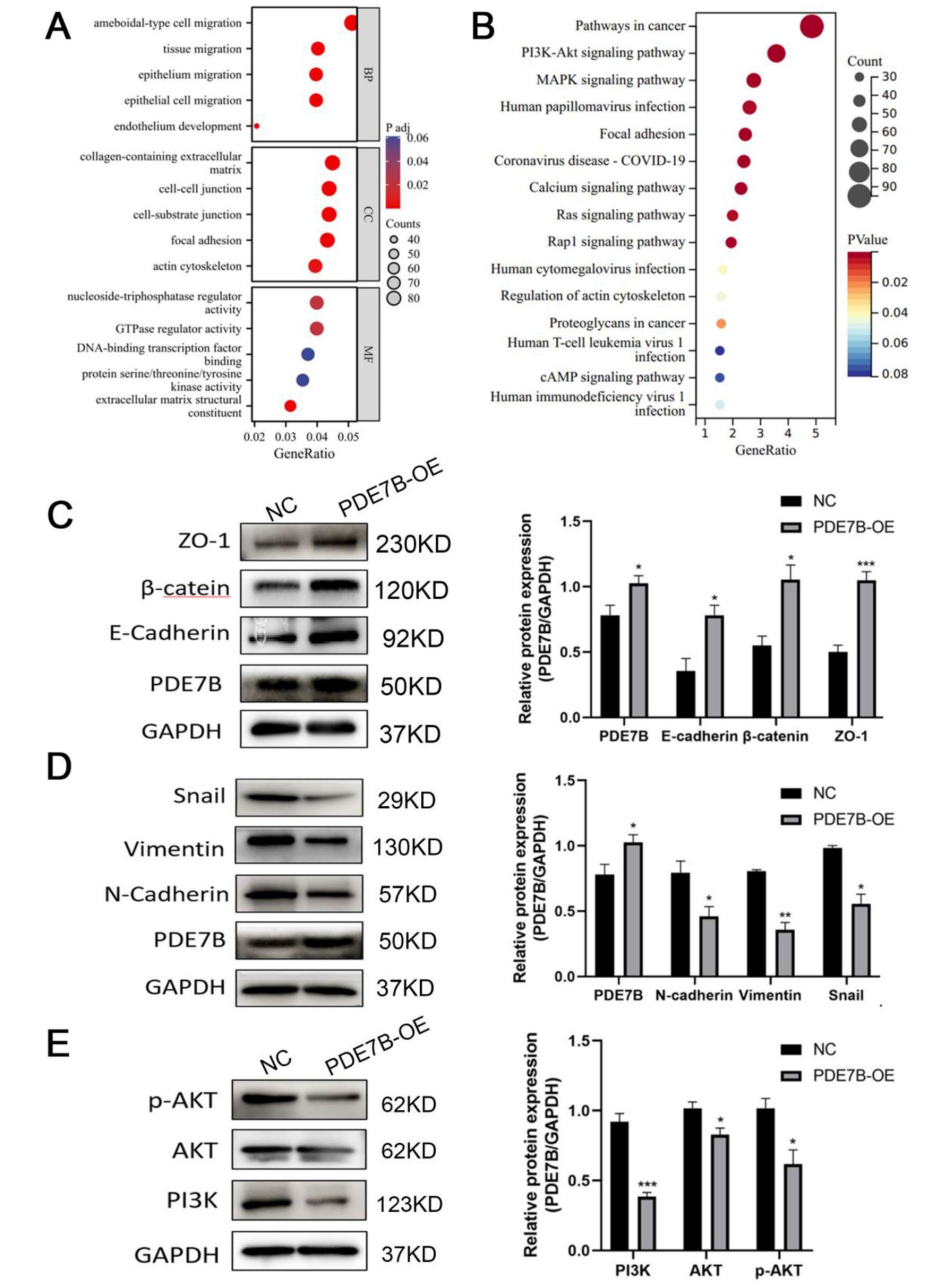
GO (**A**) and KEGG (**B**) pathway enrichment analysis of *PDE7B*-related genes in HCC; western blot to detect the expression of EMT-related markers (**C, D**) and *PI3K-AKT* signaling pathway proteins (**E**) in SK-hep-1 cell after *PDE7B* overexpression. (*, *P* < 0.05;**, *P* < 0.01;***, *P* < 0.001)

Transcriptome data of the TCGA-LIHC cohort were downloaded from the TCGA database and screened for genes associated with *PDE7B* (|r|>0.3, *P* < 0). GO enrichment analysis showed that *PDE7B*-related genes were mainly enriched in the extracellular matrix, biological processes were mainly enriched in cell migration, and molecular functions were mainly enriched in the regulation of nucleoside-triphosphate and GTPase activities (Fig. 7A). Moreover, KEGG analysis showed that these genes were mainly enriched in cancer-associated pathway, *PI3K/AKT* signaling pathway (Fig. 7B).

We selected SK-hep-1 for the validation of downstream mechanisms, and found that the protein expression of epithelial markers *E-cadherin*, *β-catenin* and *ZO-1* was significantly elevated in the PDE7B overexpression group, whereas the protein expression of mesenchymal markers *N-cadherin*, *Vimentin* and the transcription factor *Snail* was significantly reduced (Fig. 7C, D). Meanwhile, the detection revealed that the expression levels of *PI3K*, *AKT*, and *p-AKT*, the key proteins of the *PI3K/AKT* signaling pathway, were significantly reduced in the *PDE7B* overexpression group (Fig. 7E).

## Discussion

HCC is a complex disease associated with risk factors, such as HBV infection, AFP exposure, environmental factors, and genetic or epigenetic changes. DNA methylation, the most common epigenetic modification in cancer, prompts gene regulation changes that promote oncogenesis [5]. For example, CpG methylation is an epigenetic regulator of gene expression that typically results in gene silencing [7, 26]. Gaining an understanding of underlying epigenetic biomarkers can lead to a greater comprehension of the mechanisms of HCC onset and progression. Recent whole-genome sequencing technology studies have found that many genes in HCC tissues exhibit abnormal DNA methylation characteristics, and these abnormal changes can be used for subtype classifications and outcome predictions in patients with HCC [6, 27]. However, despite the recent progress, the exact role of DNA methylation in HCC remains unclear. Additionally, *PDE7B*, a member of the *PDE* family, is capable of hydrolyzing phosphate moieties and decreasing cytosolic cAMP concentrations. cAMP signaling is significant for tumor progression and treatment [28–30]. Several investigations have illustrated that the expression of *PDE7B* is dysregulated in pan-cancer [15–17, 31–33]. For instance, Brooks et al. noted that high levels of *PDE7B* expression were connected with stem cell subpopulation expansion and enhanced proliferation rate and invasiveness of tumors [15]. Zhang et al. also discovered that knockdown of *PDE7B* inhibited the proliferation and progression of triple-negative breast cancer cells [16]. *PDE7B* is also upregulated in chronic lymphocytic leukemia and set cell lymphoma and serves as a factor in the inferior survival of patients with these cancers [31–33]. Together, these findings demonstrated that *PDE7B* is predominantly an oncogene that drives the progression of tumors. However, the role of *PDE7B* in the development of HCC has not been investigated.

Within this research, we generated genome-wide DNA methylation profiles at the single-base level to identify variations in HCC tissue compared with adjacent normal tissue. In addition, we screened a series of DMRs in our HCC samples for the differentially expressed gene *PDE7B* in conjunction with transcriptomic data, and then analyzed the correlation between *PDE7B* expression and various clinical features. We also validated the expression of *PDE7B* in HCC and assessed its prognostic value using our hospital samples and online databases (TCGA, ICGC-LIRI-JP, CNHPP, GSE14520, GSE102079 and GSE107170,). We discovered that differential expression of *PDE7B* in HCC tissues was associated with HCC recurrence and that *PDE7B* was hypermethylated in HCC cells, which may be regulated by methylation of its promoter, CpG. Our results suggest that hypermethylation and downregulation of *PDE7B* are positively associated with the prognosis of HCC. In addition, *PDE7B* overexpression was found to inhibit the proliferation, migration and invasion of HCC cells in vitro, suggesting that *PDE7B* has a tumor-suppressive role in HCC.

WGBS is an effective and intuitive method for studying genome-wide DNA methylation abnormalities [34]. However, it is relatively rare to find studies to analyze HCC through the use of WGBS. In our study, We found that there were higher methylation levels in the exon and intron areas relative to the promoter areas in HCC tissues compared to adjacent normal tissues. Moreover, More widespread DNA hypomethylation was observed in HCC tissues than in normal tissues. Overall, DNA methylation occurred mainly in CG sequences, consistent with the findings of Yan et al. [27]. Furthermore, previous studies have reported global genomic DNA hypomethylation and gene-specific DNA hypermethylation or hypomethylation as epigenetic characteristics of HCC, which altered gene expression and contributed to the onset and progression of HCC [35, 36]; these findings are consistent with those of our study.

To reveal the potential functions of DMGs in HCC, we performed GO and KEGG pathway analyses of 713 hypo-DMGs and 362 hyper-DMGs. Huang et al. discovered that low capping protein (actin filament) muscle Z-line, alpha 1 (*CAPZA1*) expression drives the epithelial to mesenchymal transition by regulating actin cytoskeleton remodeling, thereby promoting HCC cell invasion and migration in vitro and in vivo [37]. In addition, numerous studies have shown that ribosomes play an important role in the development of HCC. For example, Cao et al. identified ribosome biogenesis regulator 1 (*RRS1*), a ribosomal biogenesis regulatory protein, as a key oncogene involved in HCC progression [38]. Additionally, Pang et al. identified a long non-coding RNA (*SMIM30*) that binds to ribosomal protein S6 (*RPS6)*, which regulates cell proliferation and migration to promote HCC development [39]. Han et al. found that adhesive junction-associated protein 1 (*AJAP1*) oversubscription restrained tumor initiation and pulmonary metastasis in HCC and that heterotopic expressed of *AJAP1* reduced the multiplication, oncogenicity and transfer of HCC cells [40]. Finally, Zha et al. determined that Rap1 micro RNA enhanced the sensitivity of HepG2 cells to 5-fluorouracil chemotherapy in vitro and in vivo, which promoted apoptosis [41]. Thus, all these functional enrichments observed in screened DMGs suggest an important role in the progression of HCC.

Moreover, through a combined WGBS and RNA sequencing analysis, we determined that *PDE7B* expression, a potential methylation-related tumor suppressor, was downregulated and associated with HCC prognosis. In this study, we found that *PDE7B* expression was significantly downregulated in HCC tissues and that low expression was positively associated with HCC recurrence, the serum AFP level, and survival. Additionally, the multivariate regression model identified *PDE7B* as an independent factor influencing HCC prognosis. Furthermore, *PDE7B* overexpression inhibits tumor proliferation and metastasis in vitro. Together, our results suggest that *PDE7B* may inhibit HCC progression. Sun et al. also showed that *PDE7B* was frequently downregulated in clear cell renal cell carcinoma (ccRCC) cells, which increased cell viability and migration, suggesting that *PDE7B* exerts anti-tumorigenic effects in cancers [17], consistent with our results.

DNA methylation, the most common epigenetic modification in cancer, causes gene regulation changes that promote oncogenesis [5]. DNA methylation frequently occurs in solid tumors, such as colorectal, gastric, and lung cancer [42–44]. One study demonstrated that high methylation levels of the Ras association domain-containing protein 1 (*RASSF1A*) promoter in the serum could predict early-stage HCC [45]. Additionally, Wei et al. [46] reported that the promoter region methylation status of suppressor of cytokine signaling 3 (*SOCS3*) was associated with AFP, tumor size, and the degree of tumor differentiation, serving as a biomarker for a poor HCC prognosis. In this study, we found a hypermethylated condition in the initiator area of *PDE7B* in HCC cells, and DNA methylation of *PDE7B* was negatively associated with *PDE7B* mRNA levels and HCC prognosis. Additionally, with the aid of DNA methylation sequencing, we determined the methylation of CpG islands in the initiator area of *PDE7B*, and the outcomes demonstrated that the methylation degrees of cg14623715, cg3984009 and cg04152196 loci have been amongst HCC cells than L02 cells. Finally, 5-Aza stimulation diminished the methylation level of CpG islands of the target, while enhancing the production of *PDE7B* mRNA and protein expression. Therefore, we speculate that the methylation status of *PDE7B* could be used to predict HCC progression.

Previous studies have found that abnormally frequent methylation of genes with crucial roles in regulating the cell cycle, cell death, DNA damage repair, cell proliferation, invasion, and metastasis are associated with HCC, such as *RASSF1*, adenomatous polyposis coli (*APC*), cyclin-dependent kinase inhibitor 1 A (*CDKN1A*), and cadherin-1 (*CDH1*) [45, 47, 48]. Furthermore, *PDE7B* might be a tumor suppressor in ccRCCs [17], and our study suggests that *PDE7B* could have a similar role in HCC. *PDE7B* overexpression in HCC cells inhibited cell proliferation, colony formation, and invasion. Moreover, EMT serves as one of the early markers of tumor invasion and metastasis. We found that *PDE7B* overexpression upregulated the expression of the epithelial markers *E-cadherin*, *β-catenin*, and *ZO-1*, while downregulating the expression of the mesenchymal markers *N-cadherin* and *Vimentin*, and the transcription factor *Snail*. Moreover, *PDE7B*-related genes were mainly clustered in the *PI3K-AKT* pathway, and the expression of *PI3K-AKT* pathway-related proteins decreased with the increase of *PDE7B* expression. This suggests that the increased expression of *PDE7B*, as a potential oncogene, may inhibit the EMT process of hepatocellular carcinoma cells by suppressing the *PI3K-AKT* pathway, and thus play a tumor-suppressive role.

In conclusion, the present study provides evidence that *PDE7B* has a tumor-suppressive role in HCC and that its downregulation is associated with hypermethylation of its promoter region with the ability to regulate the EMT process in HCC through the *PI3K-AKT* pathway. Based on all our findings, we suggest that *PDE7B* may present as a potential therapeutic target for the therapy of HCC.

### Electronic supplementary material

Below is the link to the electronic supplementary material.

Supplementary Material 1

Supplementary Material 2

Supplementary Material 3

## Data Availability

These sequence data have been submitted to the NCBI Sequence Read Archive (SRA) under accession number: PRJNA1103867. http://www.ncbi.nlm.nih.gov/bioproject/1103867.
