# Supplementary material for: Methylation-regulated tumor suppressor gene PDE7B promotes HCC invasion and metastasis through the PI3K/AKT signaling pathway
Source: BMC Cancer. 2024 May 22;24:624. doi: 10.1186/s12885-024-12364-w (PMC11112795; doi:10.1186/s12885-024-12364-w)
Supplement: Supplementary file 1 — Supplementary Material 1 [file 12885_2024_12364_MOESM1_ESM.docx]

**Supplementary Table 1** Clinicopathologic features of the sample

| Variable | Sample 1 | Sample 2 | Sample 3 |
| --- | --- | --- | --- |
| Tumor surgery organ | Liver | Liver | liver |
| Sex | Male | Male | Male |
| Age | 57 | 48 | 59 |
| Whether it is the primary organ | Yes | Yes | Yes |
| Whether it is metastasis | No | No | No |
| Pathological staging | Hepatocellular carcinoma | Hepatocellular carcinoma | Hepatocellular carcinoma |
| Case grading | Medium | Medium-low | Medium-high |
| Tumor size (cm) | 3.7x3 | 9x7 | 3 |
| Number of tumors | 2 | 1 | 1 |
| Cirrhotic nodules | Yes | None | Yes |
| Tumor envelope intact | Complete | Complete | Incomplete |
| T Staging | T2 | T2 | T1 |
| N Staging | N0 | N0 | N0 |
| M Staging | M0 | M0 | M0 |
| Clinical staging | Stage Ⅱ | Stage Ⅱ | Stage I |
| Time of surgery | 2020.11.04 | 2020.11.12 | 2007.6 |
| Survival time (months) | [up to now](javascript:;) | [up to now](javascript:;) | 21 |
| Duration of tumor-free survival (months) | [up to now](javascript:;) | [up to now](javascript:;) | 17 |
| HBsAg | Positive | Positive | Positive |
| HBcAb | Positive | Positive | Positive |
| AntiHCV | Negative | Negative | Negative |
| TB (umol/L) | 15.88 | 9.02 | 23.5 |
| ALT(U/L) | 92 | 43.4 | 235 |
| ALB (g/L) | 39.9 | 44.8 | 3.7 |
| AFP (ug/L) | 55.85 | >1210 | 12 |
| GGT (U/L) | 243.8 | 63.36 | 151 |

**Abbreviations:** HBsAg, hepatitis B surface antigen; HBcAb, hepatitis B core antibody; AntiHCV, hepatitis C virus antibody; TB, total bilirubin; ALT, alanine transaminase; ALB, albumin; AFP, alpha-fetoprotein; GGT, gamma-glutamyl transferase.


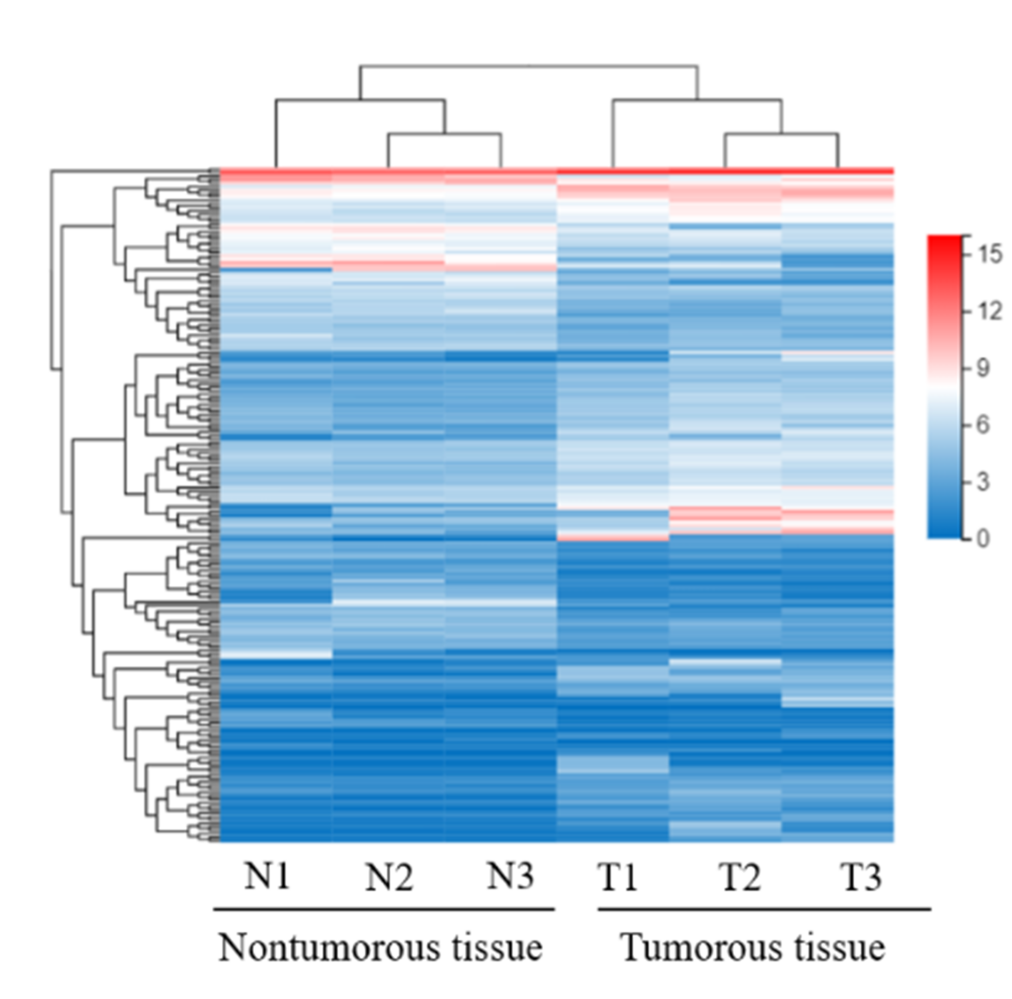


**Supplementary Fiugre 1** Heat maps of three pairs of differentially methylated genes in liver cancer tissues and adjacent tissues


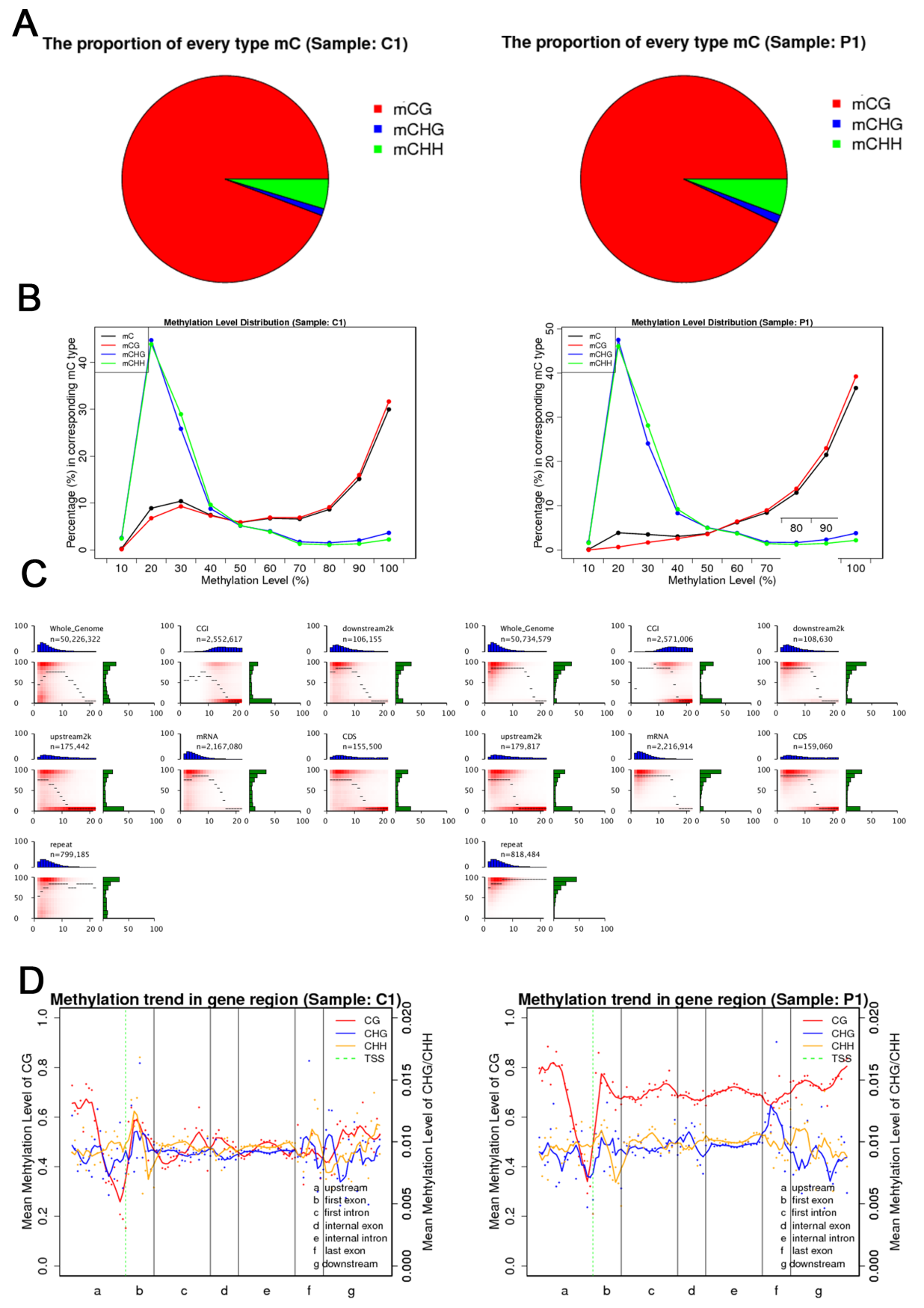


**Supplementary Fiugre 2** The proportion of every mC type in the HCC and adjacent normal (**A**); percentage of different levels of each methylation type HCC and adjacent normal (**B**); Methylation of different regions HCC and adjacent normal (**C**); Methylation trends of gene regions HCC and adjacent normal (**D**).


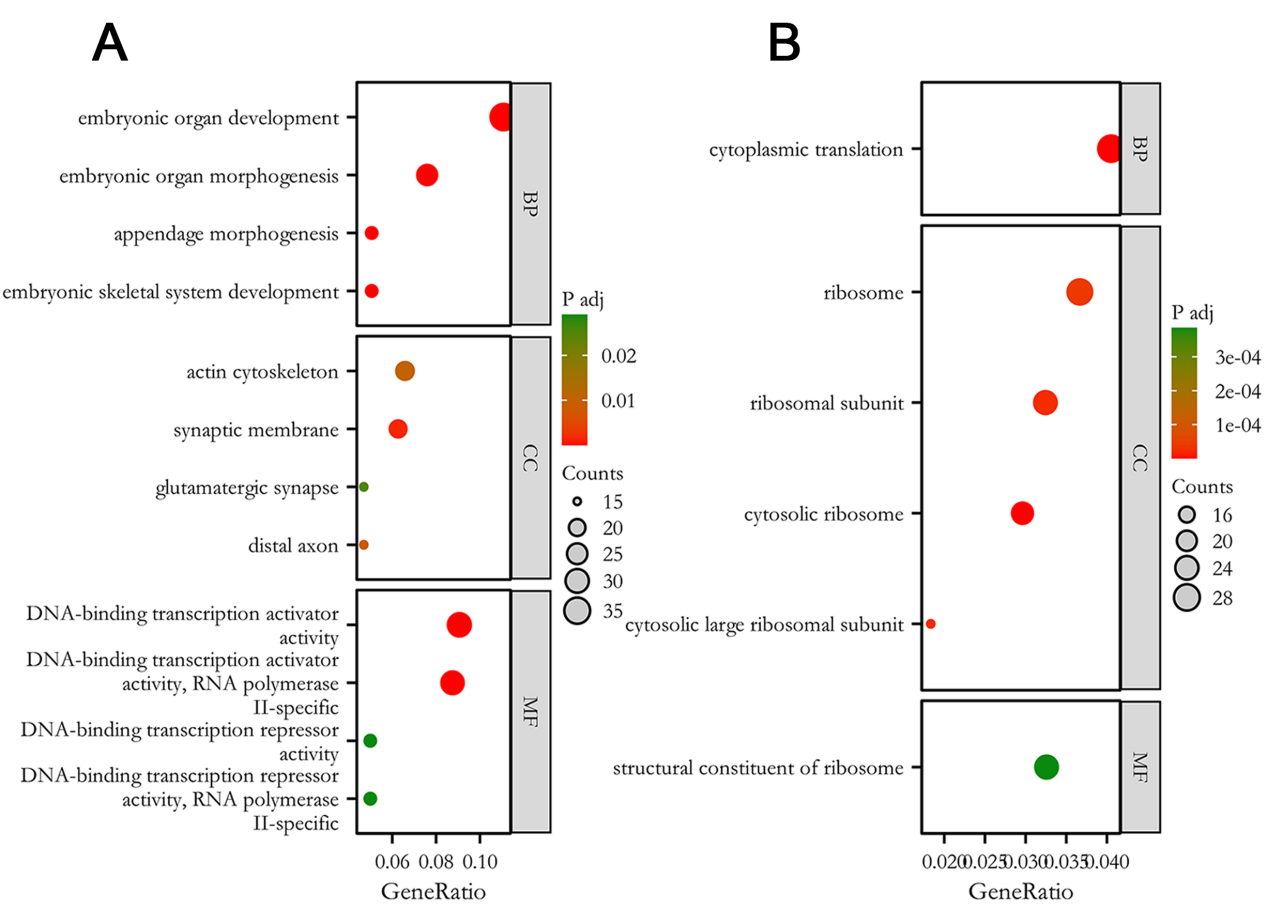


**Supplementary Fiugre 3** GO analysis of differentially hypermethylated genes (**A**); GO analysis of differentially hypomethylated genes (**B**).


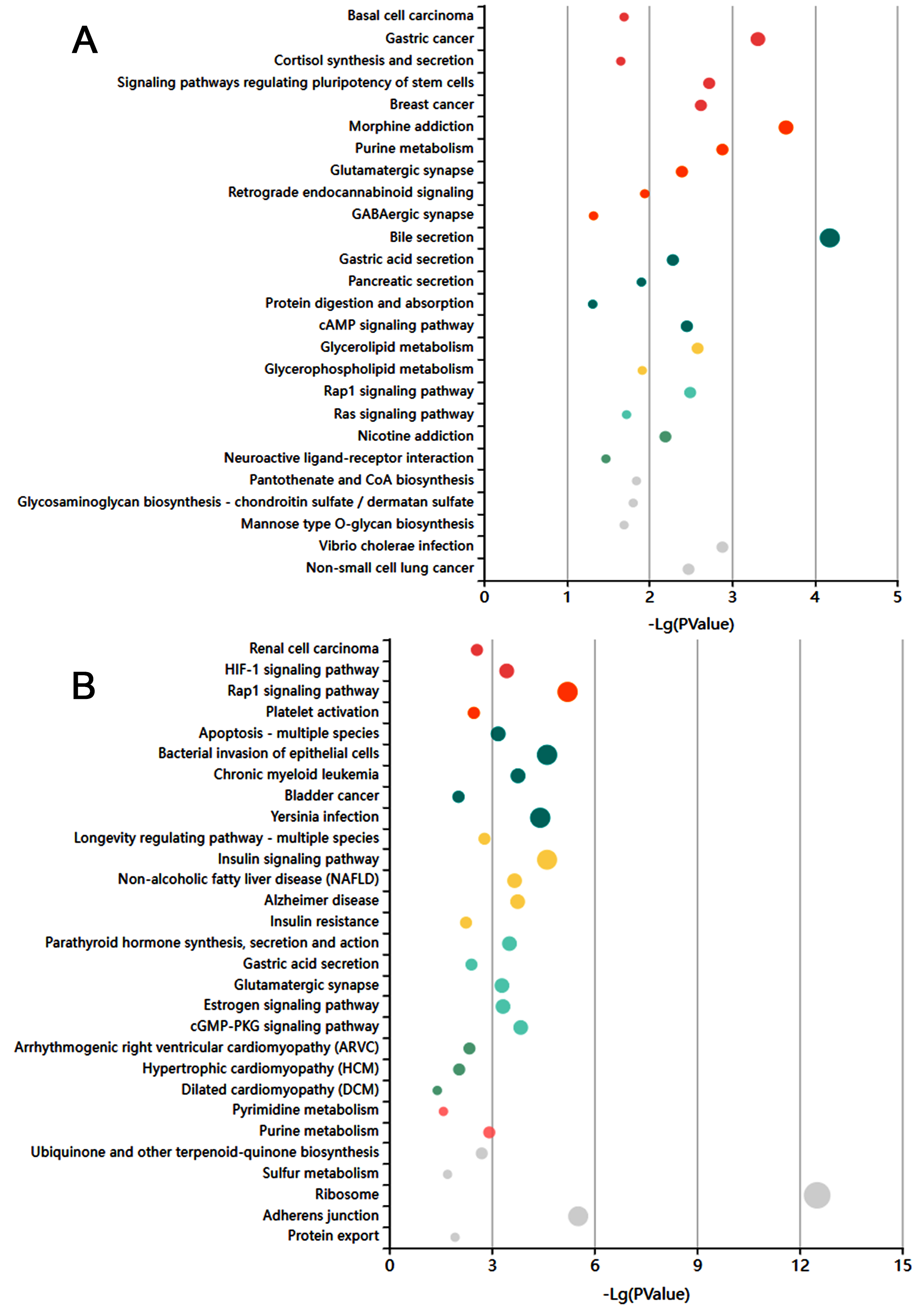


**Supplementary Fiugre 4** KEGG analysis of differentially hypermethylated genes (**A**); KEGG analysis of differentially hypomethylated genes (**B**).


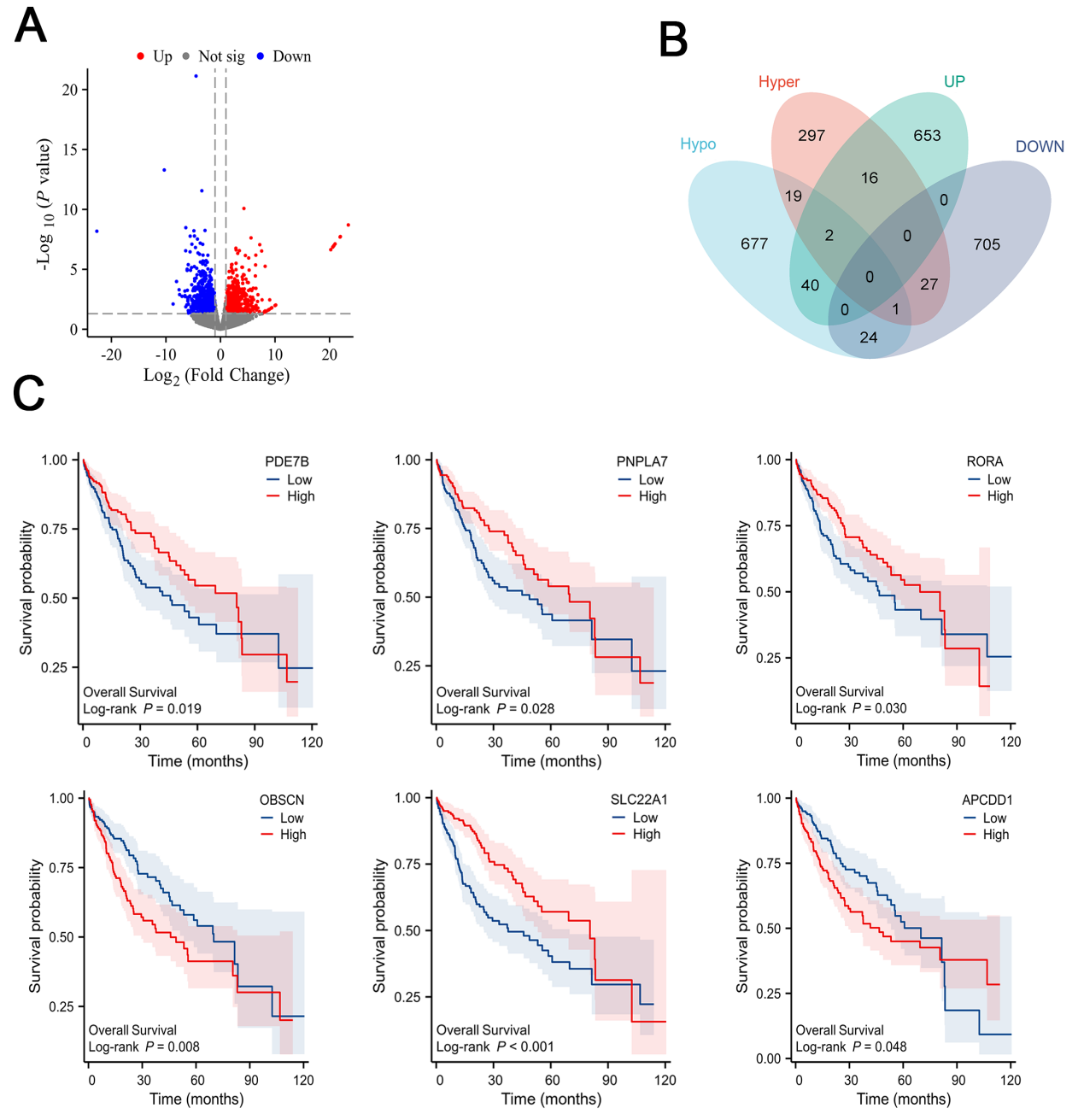


**Supplementary Fiugre 5** Volcanic maps of three pairs of differentially expressed genes in hepatocellular carcinoma and paracancer tissues (**A**); venn diagram of methylated DMR annotated genes in WGBS sequencing and differentially expressed genes in transcriptome sequencing (**B**); Survival and prognosis of HCC by six genes screened with significant expression differences (**C**).


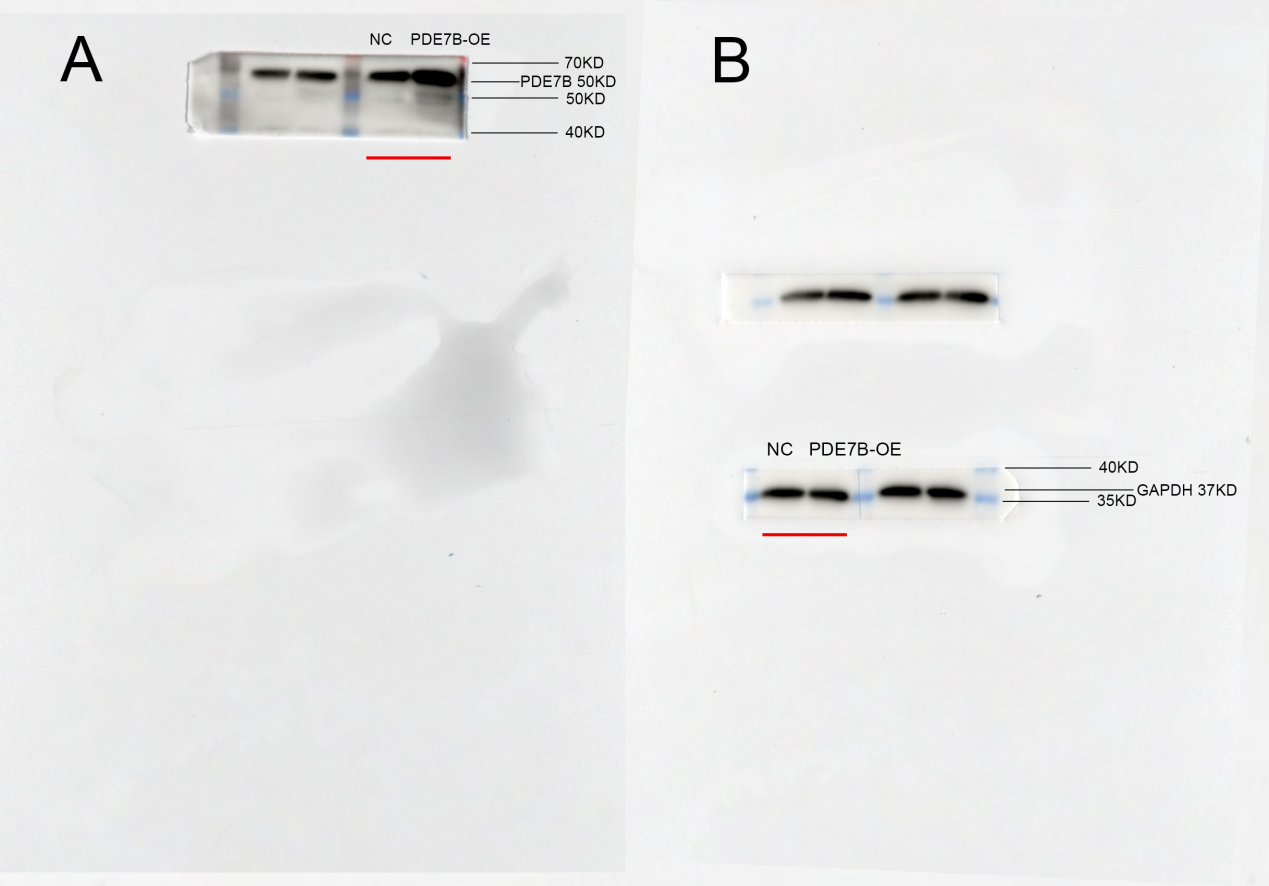


**Supplementary Fiugre 6** Raw data for westen blot in Figure 6D.


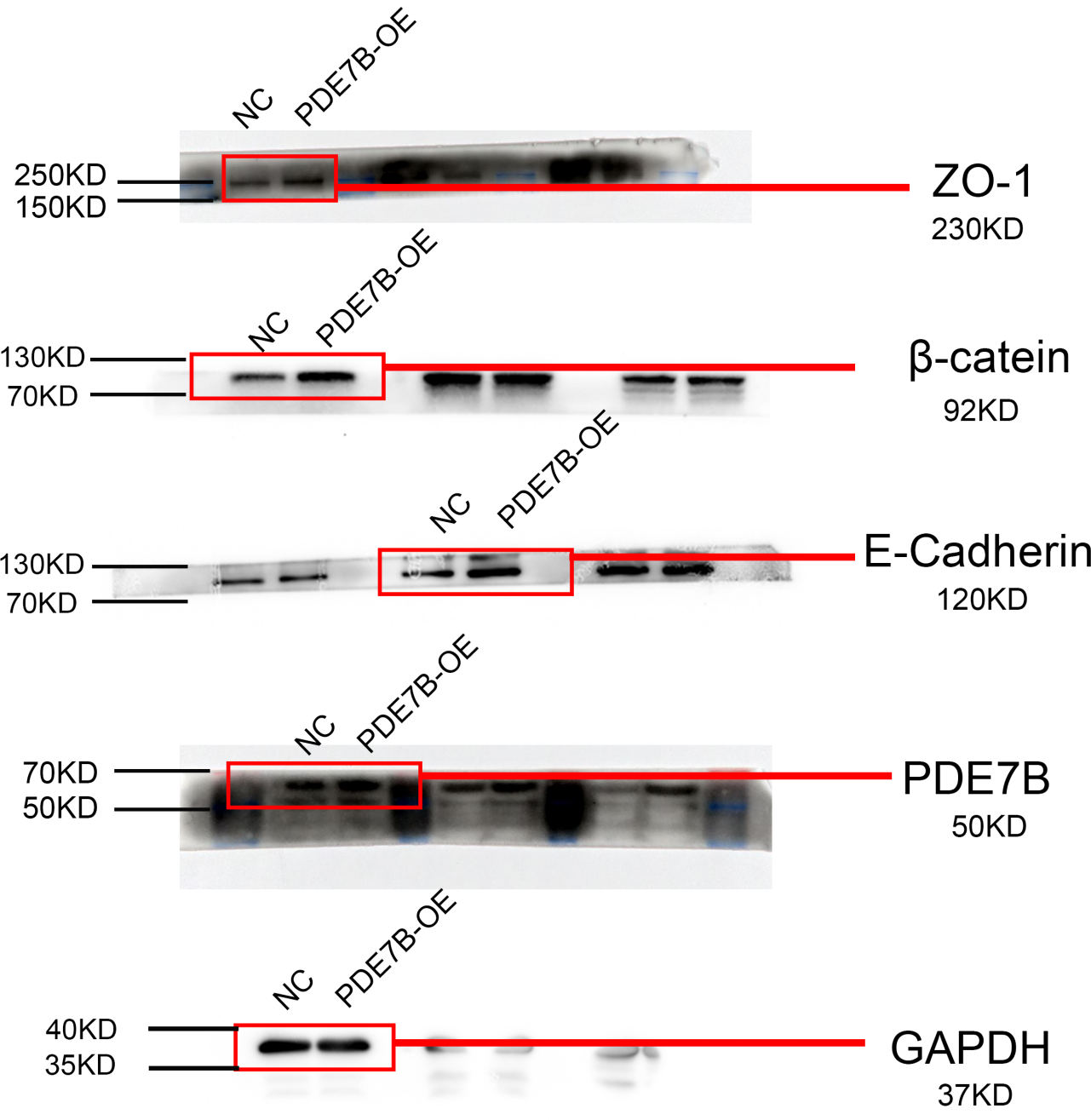


**Supplementary Fiugre 7** Raw data for westen blot in Figure 7C (The membrane edges of GAPDH are not well defined due to de-cleavage prior to antibody hybridization and a long exposure time.).


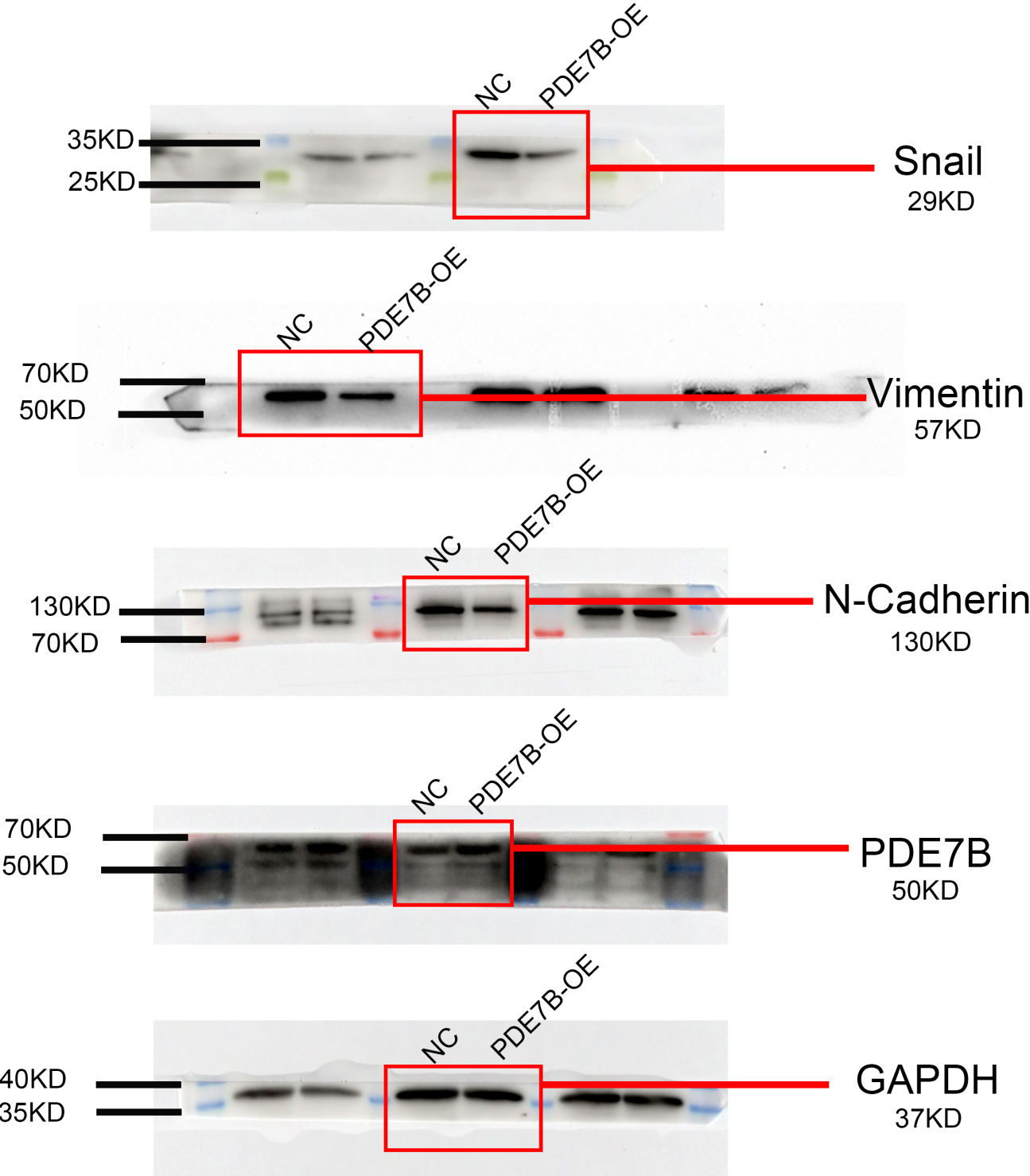


**Supplementary Fiugre 8** Raw data for westen blot in Figure 7D.


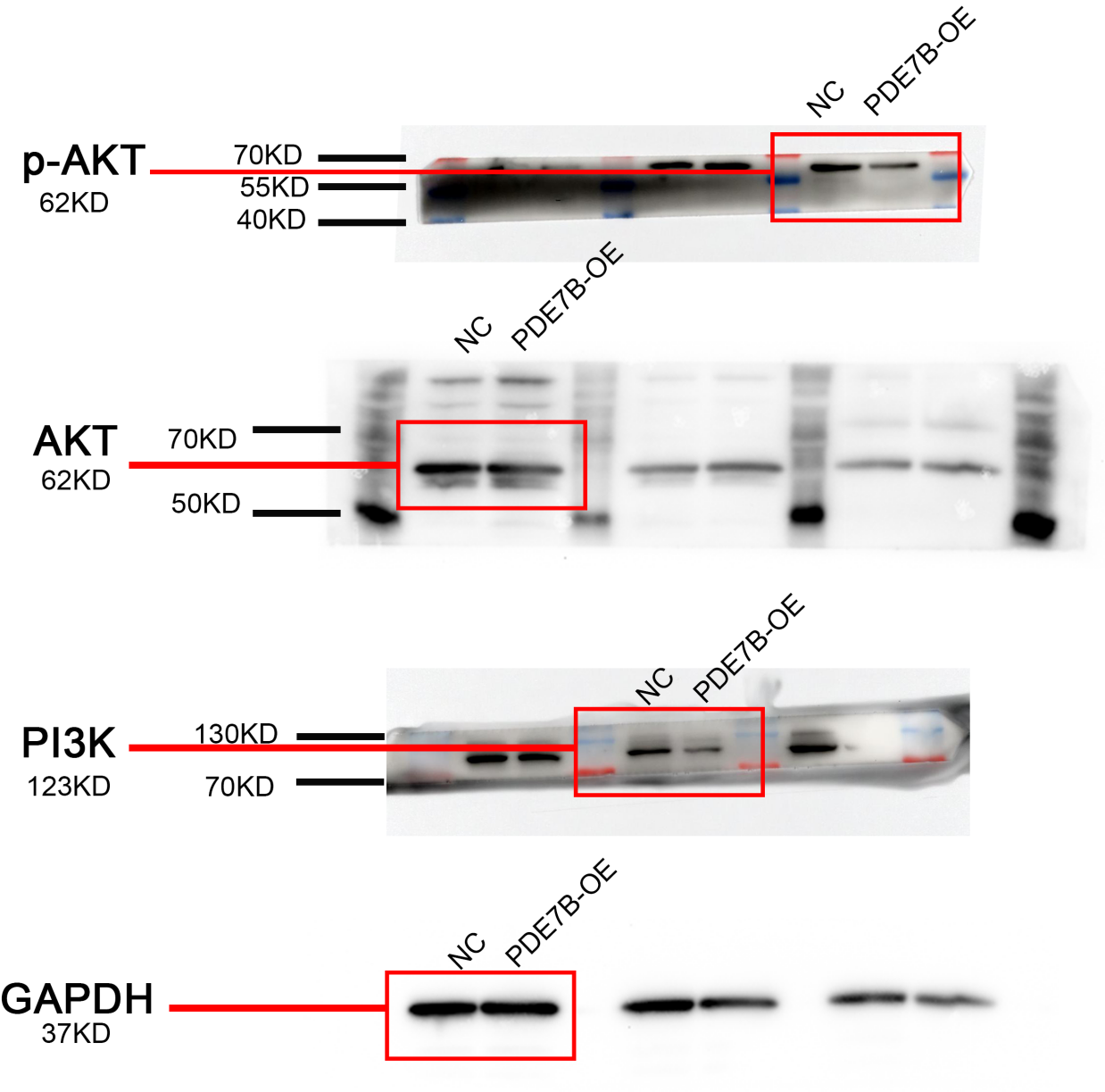


**Supplementary Fiugre 9** Raw data for westen blot in Figure 7E (The membrane edges of GAPDH are not well defined due to de-cleavage prior to antibody hybridization and a long exposure time.).
