# Supplementary material for: Methylation-regulated tumor suppressor gene PDE7B promotes HCC invasion and metastasis through the PI3K/AKT signaling pathway
Source: BMC Cancer. 2024 May 22;24:624. doi: 10.1186/s12885-024-12364-w (PMC11112795; doi:10.1186/s12885-024-12364-w)
Supplement: Supplementary file 2 — Supplementary Material 2 [file 12885_2024_12364_MOESM2_ESM.docx]

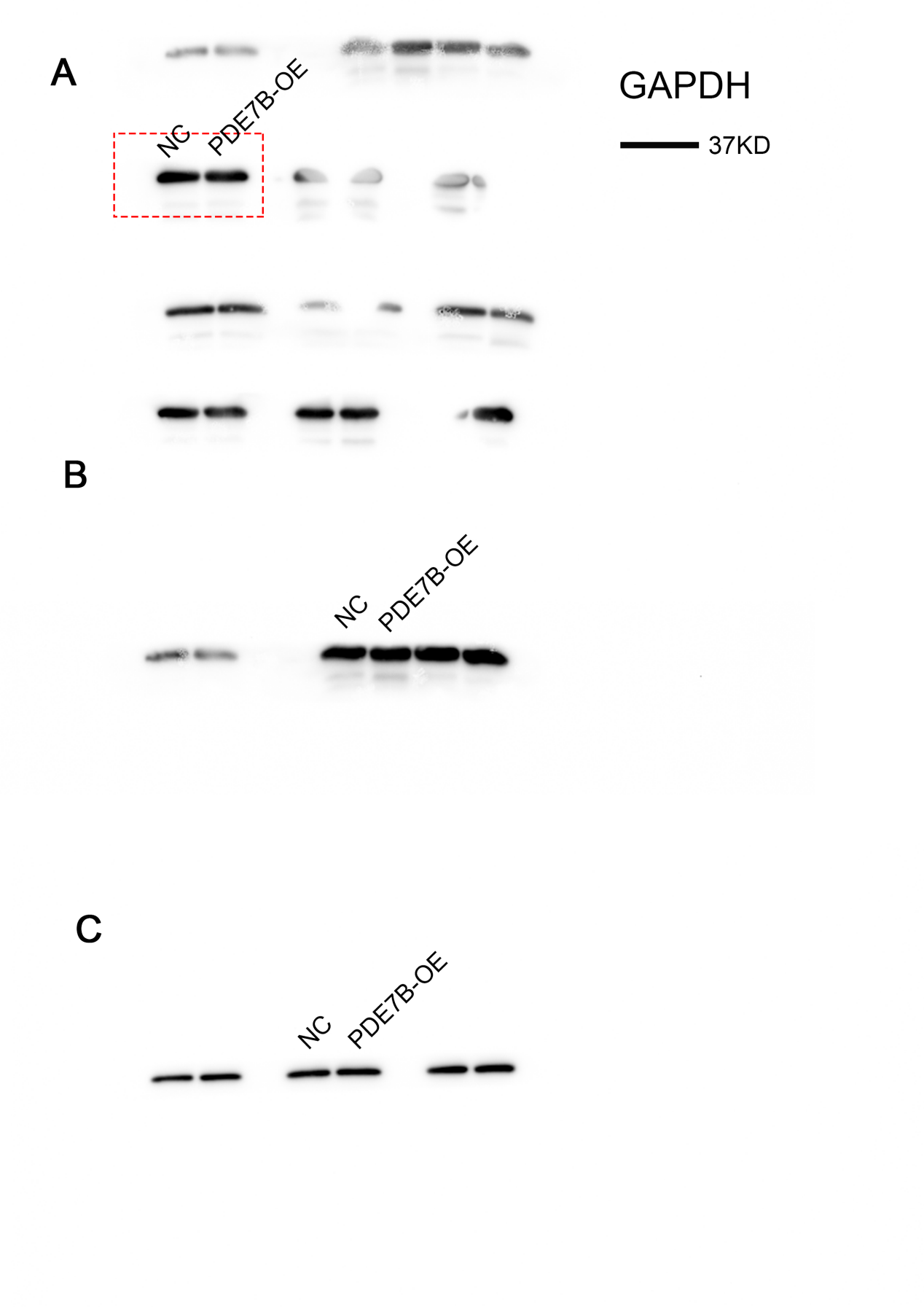


**Fig.1** Raw data for westen blot in Figure 7C GAPDH **(A)**; Multiple repetitions of the original blot map (**B,C**).


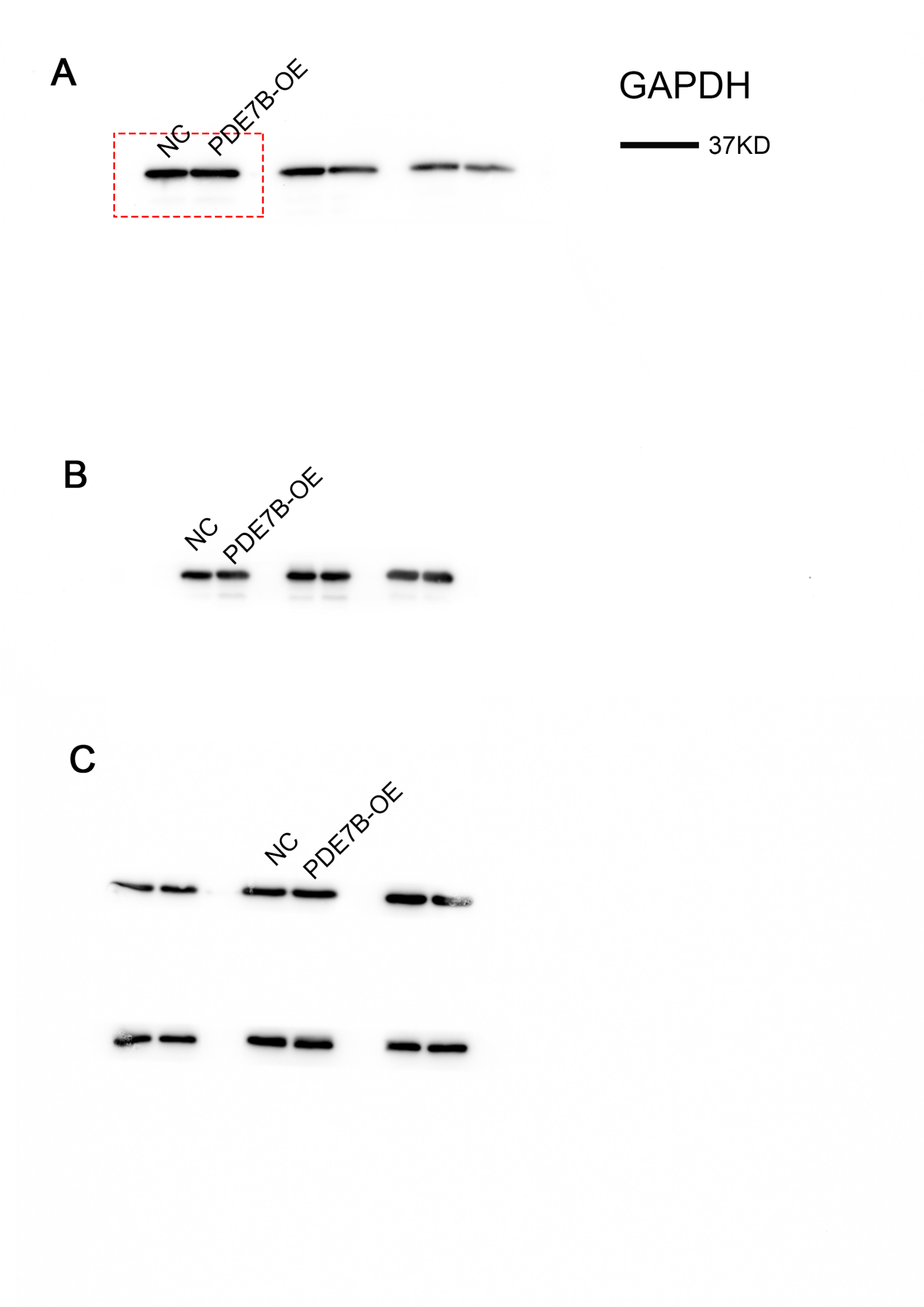


**Fig.2** Raw data for westen blot in Figure 7E GAPDH (**A**); Multiple repetitions of the original blot map (**B,C**).
