# Supplementary material for: Methylation-regulated tumor suppressor gene PDE7B promotes HCC invasion and metastasis through the PI3K/AKT signaling pathway
Source: BMC Cancer. 2024 May 22;24:624. doi: 10.1186/s12885-024-12364-w (PMC11112795; doi:10.1186/s12885-024-12364-w)
Supplement: Supplementary file 3 — Supplementary Material 3 [file 12885_2024_12364_MOESM3_ESM.docx]

**Supplementary Table 1** Primers used for RT-qPCR

| Gene | Organism | 5’-sequences-3’ |
| --- | --- | --- |
| GAPDH-F | human | ACAACTTTGGTATCGTGGAAGG |
| GAPDH-R | human | GCCATCACGCCACAGTTTC |
| PDE7B-F | human | TGGGAGATATACGACTAAGGGGT |
| PDE7B-R | human | CGGAAGTCAATGAATGGGTAGG |

**Supplementary Table 1** Western blotting antibody information

| Gene | Organism | Source | Catalog Number | Dilution |
| --- | --- | --- | --- | --- |
| GAPDH | human | Proteintech | 60004-1-Ig | 1:50000 |
| PDE7B | human | Proteintech | 17771-1-AP | 1:1500 |
| ZO-1 | human | Proteintech | 21773-1-AP | 1:1000 |
| β-catein | human | Proteintech | 17565-1-AP | 1:5000 |
| E-cadherin | human | Proteintech | 20874-1-AP | 1:5000 |
| N-cadherin | human | Proteintech | 22018-1-AP | 1:2000 |
| Snail | human | Proteintech | 13099-1-AP | 1:1000 |
| Vimentin | human | Proteintech | 10366-1-AP | 1:1000 |
| p-AKT | human | Proteintech | 66444-1-Ig | 1:5000 |
| AKT | human | Proteintech | 10176-2-AP | 1:3000 |
| PI3K | human | Proteintech | 20584-1-AP | 1:1000 |
